# Integrative multi-omics analysis identifies stellate cell-derived complement component 7 as a predictor of fibrosis and liver-related events in MASLD

**DOI:** 10.1016/j.jhepr.2026.101965

**Published:** 2026-07-22

**Authors:** Shohei Kondo, Takefumi Kimura, Takanobu Iwadare, Mohammad Taufeeq, Takao Miwa, Naoyuki Fujimori, Tomoyuki Nakajima, Shun-ichi Wakabayashi, Taiki Okumura, Hiroyuki Kobayashi, Yuki Yamashita, Satsuki Isogai, Tomoo Yamazaki, Mai Iwaya, Takeshi Uehara, Takeji Umemura, Saket Choudhary, Bernd Schnabl, Shanu Jain, Naoki Tanaka

**Affiliations:** 1 Department of Medicine, Division of Gastroenterology, Shinshu University School of Medicine, Matsumoto, Japan; 2 Consultation Center for Liver Diseases, Shinshu University Hospital, Matsumoto, Japan; 3 Institute of Biomedical Sciences, Shinshu University, Matsumoto, Japan; 4 Department of Biological Sciences and Bioengineering, Indian Institute of Technology Kanpur, Kanpur, India; 5 Department of Medicine, University of California San Diego, La Jolla, CA, USA; 6 Department of Gastroenterology/Internal Medicine, Graduate School of Medicine, Gifu University, Gifu, Japan; 7 Department of Gastroenterology, Shinshu Ueda Medical Center, Ueda, Japan; 8 Department of Laboratory Medicine, Shinshu University School of Medicine, Matsumoto, Japan; 9 Shinshu University School of Medicine, Matsumoto, Japan; 10 Koita Centre for Digital Health, Indian Institute of Technology Bombay, Mumbai, India; 11 Department of Medicine, VA San Diego Healthcare System, San Diego, CA, USA; 12 Department of Global Medical Research Promotion, Shinshu University Graduate School of Medicine, Matsumoto, Japan; 13 International Relations Office, Shinshu University School of Medicine, Matsumoto, Japan

**Keywords:** MASLD, NAFLD, Fibrosis, Liver-related events, Complement, C7, Multi-omics, Biomarker, UK Biobank

## Abstract

**Background & Aims:**

Metabolic dysfunction-associated steatotic liver disease (MASLD) is a growing global health burden, with advanced fibrosis and liver-related events (LREs) being key prognostic factors. However, existing non-invasive biomarkers insufficiently capture fibrosis severity and LRE risk. We aim at identifying a mechanistically grounded biomarker for clinical risk stratification.

**Methods:**

We performed an integrative multi-omics analysis using publicly available hepatic transcriptomic and plasma proteomic datasets together with in-house spatial transcriptomic analyses, followed by validation in three MASLD cohorts: biopsy-confirmed Japanese (n = 430), vibration-controlled transient elastography-based (n = 120), and UK Biobank (n = 4,486) cohorts. LRE risk was assessed using Kaplan-Meier and Cox regression analyses. Single-nucleus RNA sequencing, *in situ* hybridization, and *in vitro* assays using hepatic stellate cells (HSCs) were performed to assess molecular localization and functional relevance.

**Results:**

Complement component 7 (C7) was consistently identified as the top candidate. Spatial transcriptomics and *in situ* hybridization localized C7 expression to activated HSCs within fibrotic regions. Serum C7 levels strongly correlated with fibrosis stage and liver stiffness. A threshold ≥140 μg/ml predicted significantly higher LRE risk and remained independently predictive (hazard ratio 4.54, 95% confidence interval 1.49–13.82). In area under the receiver operating characteristic curve analysis, C7 outperformed FIB-4, autotaxin, AST-to-platelet ratio index (APRI), FibroScan-AST (FAST), and AST-to-ALT ratio (AAR), with AUROCs of 0.90 *vs*. 0.85, 0.84, 0.77, 0.75, 0.67, respectively). External validation in UK Biobank supported these findings. Spatial module-score analyses linked C7-enriched regions to extracellular matrix/HSC-related fibrogenic programs and progenitor/ductular reaction signatures. *C7* knockdown in LX-2 cells suppressed extracellular matrix genes including *LAMA2*, *LAMB1*, and *COL6A3*.

**Conclusions:**

C7 is a mechanistically supported, non-invasive biomarker that surpasses conventional indices in predicting fibrosis and LREs in MASLD. These findings highlight complement-mediated fibrogenesis and C7 as a potential tool for clinical risk stratification.

**Impact and implications:**

This study identifies complement component 7 (C7) as a non-invasive biomarker associated with fibrosis severity and liver-related events in MASLD through an integrative multi-omics approach. C7 was linked to HSC-rich fibrotic niches, extracellular matrix remodeling, and future liver-related risk, with external validation in the UK Biobank cohort. These findings suggest that serum C7 may improve risk stratification by capturing both fibrosis burden and clinically relevant disease progression in MASLD.

## Highlights

• Complement C7 was identified as a biomarker associated with fibrosis severity and liver-related events in MASLD.
• Serum C7 outperformed conventional non-invasive markers for detecting liver-related events.
• Spatial and single-nucleus transcriptomics linked *C7* to HSC-rich fibrotic niches.
• External validation in the UK Biobank supported the clinical relevance of C7.
• *C7* knockdown reduced extracellular matrix gene expression in LX-2 cells.

## Graphical abstract

**Figure:**
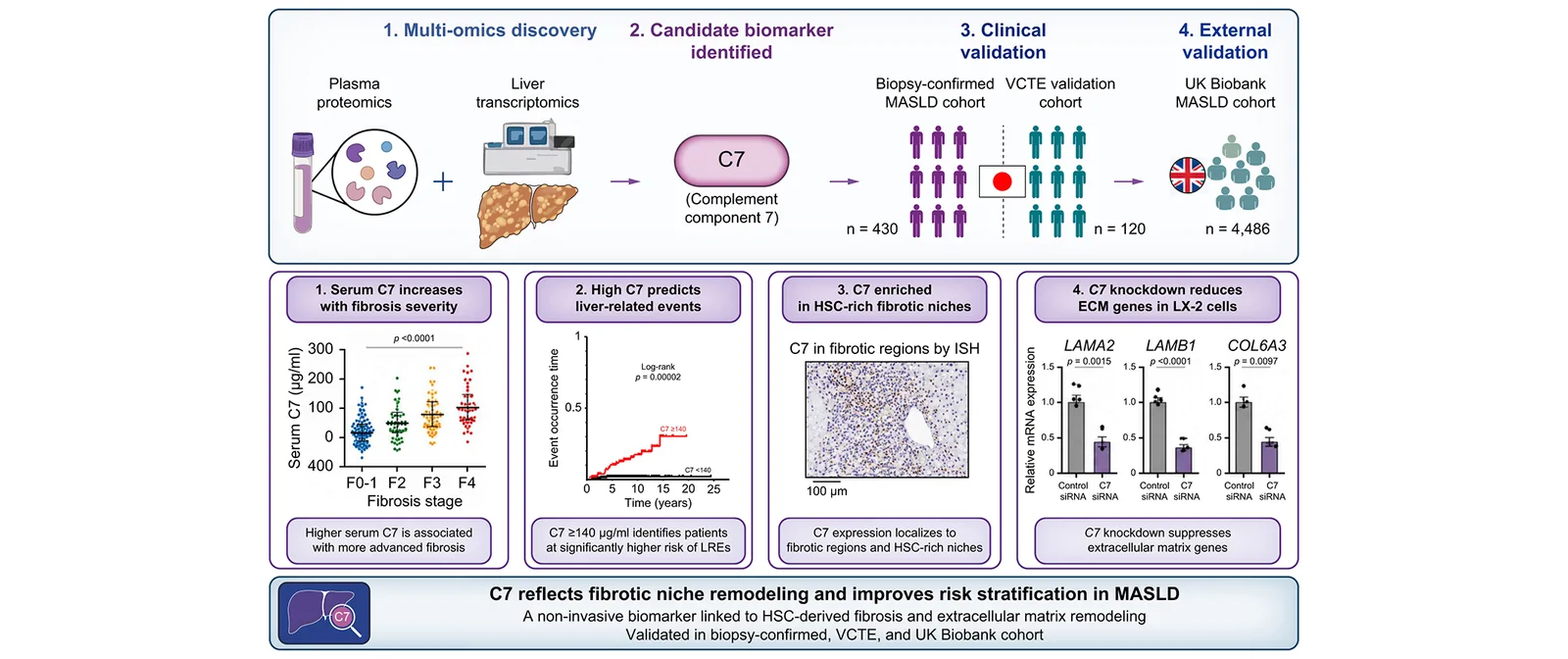

## Introduction

Metabolic dysfunction-associated steatotic liver disease (MASLD) is a prevalent chronic liver disease characterized by hepatic steatosis in the absence of excessive alcohol consumption.^1^^,^^2^ MASLD is increasingly recognized as a major cause of liver-related morbidity and mortality worldwide.^3^^,^^4^ Hepatic fibrosis severity and liver-related events (LREs), including hepatocellular carcinoma (HCC) and liver failure, are key prognostic factors in MASLD.^5^^,^^6^ Accordingly, accurate risk stratification is essential for predicting and monitoring these primary clinical outcomes of concern.

Current non-invasive biomarkers and imaging modalities have limitations in predicting disease progression and LRE risk in MASLD.^7^ Although liver biopsy remains the gold standard for fibrosis assessment, its invasiveness, sampling variability, and cost limit its clinical utility.^8^ Non-invasive alternatives, such as vibration-controlled transient elastography (VCTE) and serum biomarkers (*e.g.* fibrosis-4 [FIB-4] index and non-alcoholic fatty liver disease [NAFLD] fibrosis score), offer limited accuracy, particularly for intermediate fibrosis stages.^9^^,^^10^ Moreover, these methods primarily assess fibrosis severity but fail to directly predict LREs, a critical determinant of patient outcome.

To address these limitations, we conducted an integrative multi-omics analysis using publicly available hepatic transcriptomic and plasma proteomic datasets together with in-house spatial transcriptomics to identify novel prognostic biomarkers in MASLD. The identified candidate was then validated in two independent MASLD cohorts and further examined in the UK Biobank cohort, whereas single-nucleus RNA sequencing (snRNA-seq), *in situ* hybridization (ISH), and cell experiments were used to assess cellular localization and potential contribution to fibrogenesis (Fig. 1A). These complementary approaches identified complement component 7 (C7) as the most consistently supported candidate across the different analytical layers of the study and as a strong predictor of fibrosis progression and LRE risk in patients with MASLD.

**Fig. 1:**
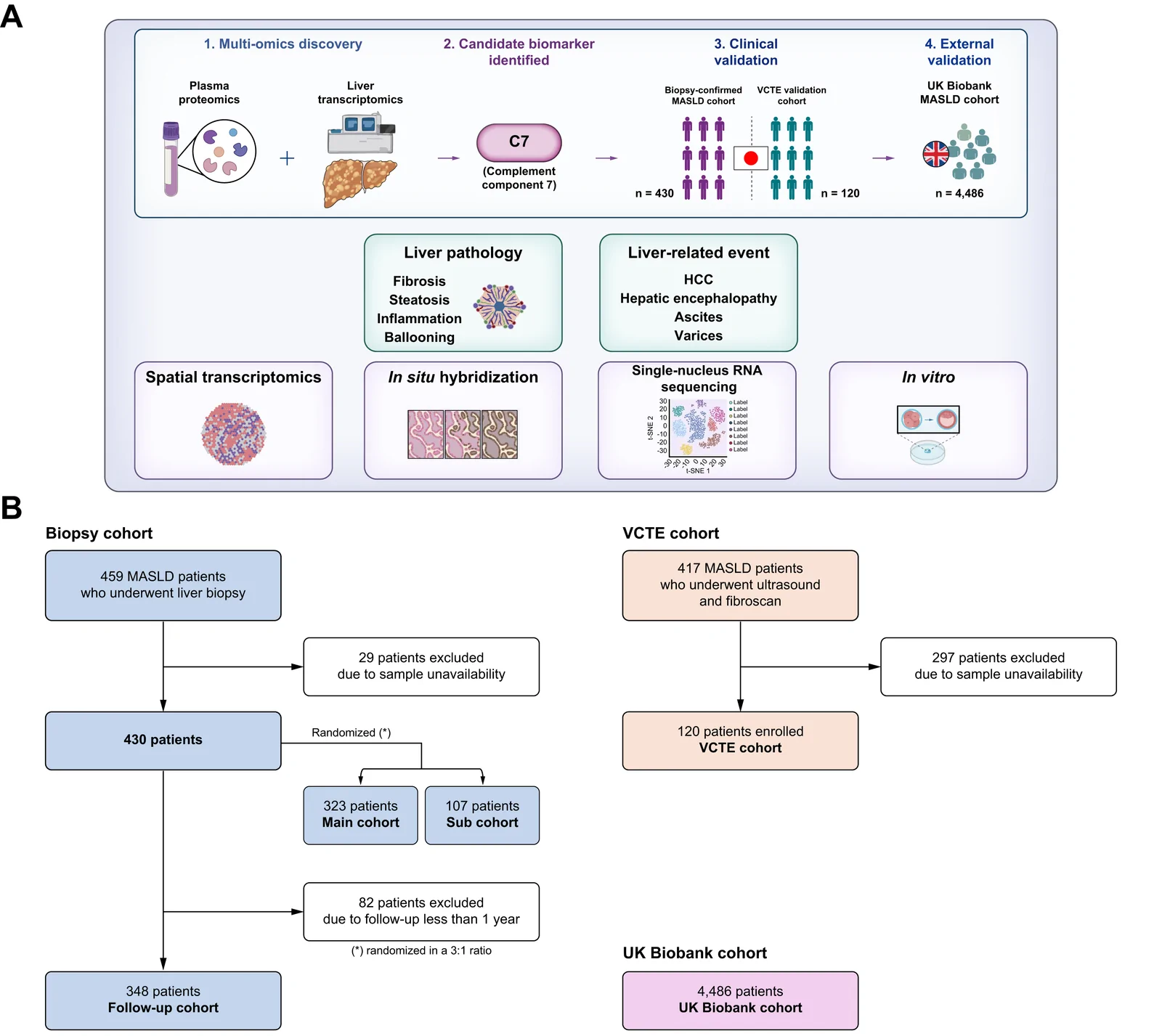
Study overview and cohort design. (A) Overview of study workflow. This study integrated plasma proteomics and liver transcriptomics to identify candidate proteins associated with fibrosis and liver-related events in MASLD. Complement component 7 (C7) was selected as the lead biomarker candidate. The performance of C7 was evaluated using a biopsy-proven MASLD cohort and validated in an independent VCTE cohort. Additional analyses included spatial transcriptomics, *in situ* hybridization, single-nucleus RNA sequencing, and *in vitro* experiments to elucidate the mechanistic role of C7 in liver pathology and clinical outcomes. (B) Study cohorts and patient selection. Biopsy cohort: A total of 459 patients with MASLD who underwent liver biopsy were initially included. Of these, 29 patients were excluded due to sample unavailability, leaving 430 patients for fibrosis biomarker evaluation. These patients were randomly assigned at a 3:1 ratio to the Main cohort (n = 323) for biomarker identification and the Sub cohort (n = 107) to assess biomarker performance. Additionally, 348 patients with at least 1 year of follow-up were included in the Follow-up cohort, and 82 patients were excluded due to insufficient follow-up duration (<1 year). VCTE cohort: A separate cohort of 417 patients with MASLD underwent ultrasound and VCTE assessment. Owing to sample unavailability, 297 patients were excluded, resulting in 120 patients enrolled in the VCTE cohort, which served as an independent validation cohort for fibrosis biomarker findings. UK Biobank cohort: A total of 4,486 participants with MASLD and available plasma C7 measurements were included as an external validation cohort. MASLD, metabolic dysfunction-associated steatotic liver disease; VCTE, vibration-controlled transient elastography.

## Materials and methods

### Patients and clinical examinations

This retrospective study, including its cohorts, was approved by the Committee for Medical Ethics of Shinshu University School of Medicine (Matsumoto, Japan; no. 5797) and conducted in accordance with the Declaration of Helsinki 1975 (revised in 1983). Written informed consent was obtained from all participants.

A total of 459 Japanese patients with MASLD who were admitted to Shinshu University Hospital (Matsumoto, Japan) between January 1998 and May 2022 and underwent liver biopsy were initially enrolled. MASLD was suspected based on the presence of hepatorenal contrast and increased hepatic echogenicity on abdominal ultrasonography, average daily ethanol consumption <20 g, and the exclusion of other causes of liver dysfunction, including viral hepatitis, drug-induced liver injury, autoimmune liver disease, primary sclerosing cholangitis, Wilson’s disease, hereditary hemochromatosis, and citrin deficiency.^11^^,^^12^

The study design and cohort classifications are illustrated in Fig. 1. Among the liver biopsy-confirmed MASLD cases (n = 459), 430 patients with available serum samples were included for fibrosis biomarker evaluation. These patients were randomly assigned at a 3:1 ratio to the Main cohort (n = 323) for biomarker identification and the Sub cohort (n = 107) to assess biomarker performance. In addition, 348 patients with at least 1 year of follow-up data were included in the Follow-up cohort to track disease progression. Our findings were further validated in an independent validation cohort (VCTE cohort; n = 120) to confirm robustness.

Hypertension (HT) was defined as systolic blood pressure >140 mmHg, diastolic blood pressure >90 mmHg, or the use of antihypertensive medications.^13^ Dyslipidemia (DL) was diagnosed based on total cholesterol level ≥220 mg/dl, LDL cholesterol ≥140 mg/dl, triglycerides ≥150 mg/dl, or the use of lipid-lowering medications.^14^ Diabetes mellitus (DM) was defined as fasting blood glucose level ≥126 mg/dl, hemoglobin A1c ≥6.5%, or the use of insulin or oral hypoglycemic agents.^15^ Body weight and height were measured in a fasting state before liver biopsy, and all laboratory data were collected on the same day. Serum C7 levels were measured using a commercial ELISA kit (Complement C7, Human, ELISA Kit; Hycult Biotech, Uden, the Netherlands) according to the manufacturer’s instructions. Serum C5a and soluble C5b-9 levels were measured using commercial ELISA kits (Human Complement C5a ELISA Kit and Human Complement C5b-9 [soluble] ELISA Kit; Invitrogen, Carlsbad, CA, USA) according to the manufacturers’ instructions.

### VCTE measurement

Patients for VCTE evaluation were recruited between September 2014 and March 2024. Liver stiffness measurement (LSM) was performed using FibroScan® (Echosens, Paris, France) following the manufacturer’s guidelines.^16^ Examinations were conducted by experienced operators blinded to clinical and histological data. The procedure was performed in a fasting state, with patients lying supine and the probe placed intercostally over the right liver lobe at the midaxillary line. At least 10 valid measurements were obtained per patient, and the median LSM value was used for analysis. To ensure measurement reliability, the IQR-to-median ratio was required to be ≤30%. The FibroScan-AST (FAST) score was calculated from LSM, controlled attenuation parameter, and AST according to the published method.^17^

### Histological examinations

Liver biopsy specimens measuring at least 1.5 cm in length were obtained from segments 5 or 8 using a 14-gauge needle, and then immediately fixed in 10% neutral formalin. Sections were cut at a thickness of 4 μm and stained with H&E and Azan-Mallory. Liver histology was assessed by an independent expert pathologist (MI) in a blinded manner according to the NAFLD activity scoring (NAS) system.^18^^,^^19^ Steatosis was graded on a scale of 0 to 3 based on the proportion of hepatocytes containing lipid droplets, with <5% classified as grade 0, 5–33% as grade 1, >33–66% as grade 2, and >66% as grade 3. Lobular inflammation was also scored on a 0 to 3 scale based on the number of inflammatory foci observed per 200x field, with no foci classified as grade 0, <2 foci as grade 1, 2–4 foci as grade 2, and >4 foci as grade 3. Hepatocellular ballooning was evaluated on a scale of 0 to 2, with no ballooned hepatocytes classified as grade 0, few as grade 1, and many as grade 2. Calculated as the sum of the scores for steatosis (0–3), lobular inflammation (0–3), and hepatocellular ballooning (0–2), the NAS score ranged from 0 to 8 and was used to assess disease activity. Fibrosis stage (F) was classified into five categories: F0 (no fibrosis), F1 (perisinusoidal or periportal fibrosis), F2 (perisinusoidal and portal/periportal fibrosis), F3 (bridging fibrosis), and F4 (cirrhosis).

### Patient follow-up

Patients were regularly monitored at intervals of at least 12 months with the inclusion of ultrasonography or computed tomography. Serum alpha-fetoprotein (AFP) levels were measured at each assessment. For patients with cirrhosis (F4), imaging and laboratory evaluations were conducted at least every 6 months. HCC was diagnosed according to the American Association for the Study of Liver Diseases guidelines, which define HCC as a hepatic lesion ≥2 cm in diameter showing a typical vascular pattern of arterial phase hyperenhancement with subsequent washout in the portal venous or delayed phases on a single dynamic imaging modality.^20^ For lesions measuring 1–2 cm, this diagnosis required confirmation of a typical vascular pattern on two dynamic imaging modalities. AFP levels were used as a supportive marker but not as a sole diagnostic criterion.^20^

In the Follow-up cohort, LREs included the development of HCC, grade II or higher hepatic encephalopathy, poorly controlled ascites requiring hospitalization, and esophagogastric varices requiring endoscopic treatment (ligation, sclerotherapy, or balloon-occluded retrograde transvenous obliteration), including cases of variceal rupture.^21^ The follow-up period was defined as the duration from MASLD diagnosis to the occurrence of an LRE or the last recorded follow-up visit. If no events occurred, the follow-up period was determined by protocol surveillance confirming the absence of LREs. Conversely, LRE status reflected the presence of current or previous LREs at the time of MASLD diagnosis rather than newly developed events during the observation period in the VCTE cohort.

### External validation in the UK Biobank cohort

External validation was conducted using the UK Biobank cohort. The UK Biobank study was approved by the North West Multi-centre Research Ethics Committee (REC reference: 11/NW/0382), and the present analysis was conducted under approved application number 930515. All participants provided written informed consent.

Eligible participants with MASLD and available C7 measurements were identified after applying predefined exclusion criteria. Plasma C7 levels were measured using the Olink® Explore platform and expressed as normalized protein expression values on a log_2_ scale. Major adverse liver outcomes (MALOs) were defined as incident ascites, cirrhosis, varices, HCC, liver failure, liver transplantation, and other major liver outcomes during follow-up. Detailed eligibility criteria, definition of MASLD, outcome codes, alcohol intake assessment, and handling of missing data are provided in Fig. S1A and Table S8–S12.

### Transcriptomics analysis

Gene expression analysis was conducted using publicly available microarray data from GSE49541.^22^^,^^23^ Raw expression data were analyzed with GEO2R, an interactive web tool for differential expression analysis of Gene Expression Omnibus (GEO) datasets. Data were normalized using quantile normalization, and differentially expressed genes among fibrosis stages were identified using the limma package. Significance thresholds were respectively set at |log_2_ fold change| ≥1 and an adjusted *p* value of <0.05 based on Benjamini-Hochberg false discovery rate (FDR) correction.

### Proteomics analysis

Proteomics data were obtained from the Proteome-Phenome Atlas, an open-access database providing curated proteomics datasets.^24^^,^^25^ Differential protein expression analysis was conducted using linear models implemented in the limma package, and statistical significance was determined using adjusted *p* values based on Benjamini-Hochberg FDR correction. Protein quantification was performed using label-free quantification, which allowed for relative protein abundance comparisons across samples. This approach enabled the identification of differentially expressed proteins associated with fibrosis severity.

### Spatial transcriptomics

Spatial transcriptomics was performed to map gene expression within liver tissue sections. Tissue samples were freshly frozen, sectioned at a thickness of 10 μm using a cryostat, and mounted onto specialized capture slides. The sections were then fixed with cold methanol and stained with H&E to visualize tissue morphology. High-resolution images of the stained sections were captured for reference and alignment. Gene expression was determined using spatially barcoded oligonucleotide arrays on each slide. RNA molecules from each spot were reverse transcribed to generate spatially encoded complementary DNA (cDNA), which was subsequently used to construct cDNA libraries following the manufacturer’s protocol (10x Genomics, Pleasanton, CA, USA). The libraries were sequenced on the Illumina NovaSeq 6000 platform (Illumina, San Diego, CA, USA) while ensuring sufficient read depth for comprehensive gene expression profiling across the tissue sample.

Raw sequencing data were processed using Space Ranger (10x Genomics, Pleasanton, CA, USA) to align spatial barcodes with the corresponding gene expression data. The resulting spatial gene expression matrix was normalized, and then analyzed using Loupe Browser (10x Genomics, Pleasanton, CA, USA) to assess differential gene expression across tissue regions. Colocalization and spatial gene expression patterns were further examined to identify region-specific transcriptomic profiles.

To characterize the spatial microenvironment associated with *C7*-enriched regions, module-score analyses were performed using predefined gene sets representing extracellular matrix (ECM)/hepatic stellate cell (HSC)-related fibrogenic programs, hypoxia-related signatures, and progenitor/ductular reaction programs. Genes were selected based on biological relevance to each program and detectability in the spatial transcriptomic dataset. For each spatial spot, module scores were calculated as the average normalized expression of detected genes in each module after z-score standardization. Detailed gene sets are provided in the Supplementary Methods.

### snRNA-seq analysis

We performed snRNA-seq analysis to investigate the expression patterns of *C7* in MASLD-associated cirrhosis compared with healthy liver tissue. The GSE174748 dataset was downloaded from the GEO database,^26^ which included single-nucleus transcriptomic profiles from patients with non-alcoholic steatohepatitis (NASH) cirrhosis and healthy controls. In this analysis, NASH cirrhosis was referred to as MASLD cirrhosis to align with current disease terminology.

The Seurat object provided in the GEO repository was used directly in measurements. This object was already integrated, and included annotated cell types from MASLD cirrhosis (n = 2) and healthy (n = 2) liver tissues. Quality control was conducted using the Seurat R package (version 5.1.0).^27^^,^^28^ Low-quality nuclei were excluded based on the number of unique molecular identifiers (nCount_RNA), the number of detected genes per nucleus (nFeature_RNA), and the percentage of mitochondrial transcripts (percent.mito). Specifically, nuclei with nCount_RNA or nFeature_RNA values outside the 0.5–99.5% quantile range or with percent.mito readings of >10% were removed. After filtering, the dataset was normalized using Seurat’s LogNormalize() function and scaled to facilitate downstream comparisons. In the provided metadata, HSCs were annotated as “mesenchymal cells”; these labels were manually revised, and all mesenchymal cells were reclassified as HSCs.^29^ *C7* expression levels were analyzed across all annotated cell types, with particular focus on endothelial cells and HSCs. We generated violin plots using the geom_violin() function to visualize expression levels, and statistical comparisons were conducted using the Wilcoxon rank-sum test.

### Gene correlation and pathway enrichment analysis

To identify genes with expression patterns correlated with *C7*, Pearson correlation coefficient analysis across all genes in the dataset was performed. For each gene, the correlation coefficient was calculated against *C7* expression using the cor() function in R (R Foundation for Statistical Computing, Vienna, Austria). Genes were then ranked based on their correlation coefficients, and the top 1,000 *C7*-correlating genes were selected for further pathway enrichment analysis. This approach enabled the identification of genes potentially correlated with *C7* or involved in similar biological processes.

The top 1,000 genes positively correlated with *C7* expression were subjected to pathway enrichment analysis using Metascape, a comprehensive gene list annotation and analysis resource that integrates multiple bioinformatics tools.^30^^,^^31^ Pathway enrichment analysis was conducted across multiple databases including Gene Ontology (GO) Biological Process, Kyoto Encyclopedia of Genes and Genomes (KEGG), and Reactome.[32], [33], [34] For the analysis, the complete gene set detected in our snRNA-seq data was used as the background gene list to ensure appropriate statistical context for enrichment calculations. This was specified in Metascape as the "BACKGROUND" feature during gene list submission. The analysis identified significantly enriched pathways (*p* <0.05) associated with *C7*-correlated genes to understand the biological processes potentially regulated by or connected to *C7* expression in MASLD cirrhosis.

### Enrichment analysis of gene co-expression networks

To elucidate the biological significance of *C7* expression, an enrichment analysis was performed using gene co-expression data derived from snRNA-seq. Genes highly co-expressed with *C7* were identified by calculating correlation coefficients, and the top 1,000 positivelyco-expressed genes were selected for further analysis.

Functional pathway enrichment examination was conducted using Metascape.^30^ The selected genes were analyzed for enrichment in biological processes, cellular components, and molecular functions using the GO database. Additionally, pathway-level associations were assessed using the KEGG and Reactome databases. Statistical significance was determined using an adjusted *p* value of <0.05.

We generated network diagrams to visualize the relationships among enriched pathways, with clustering algorithms applied to group functionally related pathways. These analyses aimed to elucidate the potential functional roles of *C7*-associated gene networks in liver fibrosis and MASLD progression.

### *In situ* hybridization

The spatial distribution of *C7* transcripts was assessed using ISH with RNAscope® Target Probe - Hs-C7 (ADC534791; Cosmo Bio Co., Ltd., Tokyo, Japan).^35^ Liver tissue sections were fixed in 4% paraformaldehyde and hybridized with labeled probes targeting *C7*. Signal detection was performed with a chromogenic detection system, and images were acquired using a light microscope. The expression of *C7* mRNA was semi-quantitatively evaluated using a previously reported five-grade scoring system.^36^ Positive staining was defined as brown punctate signals localized in the nucleus and/or cytoplasm. The scoring criteria were as follows: score 0, no staining; score 1, one to three dots per cell; score 2, four to 10 dots per cell with no or minimal dot clusters; score 3, >10 dots per cell with <10% of total cells being positive; and score 4, >10 dots per cell with ≥10% of cells showing dot clusters. For each case, representative high-power fields (400x magnification) were selected, and all detectable nuclei within the field were evaluated. Expression levels were further categorized into negative (score 0) and positive (scores 1–4) for subsequent analysis.

### Quantitative PCR analysis

Total RNA was extracted from liver biopsy samples and cells using the RNeasy Mini Kit (Qiagen, Hilden, Germany).^37^ We synthesized cDNA with the PrimeScript RT Reagent Kit (Takara Bio, Kusatsu, Shiga, Japan) following the manufacturer’s instructions. Quantitative PCR (qPCR) was performed using SYBR™ Green Master Mix (Applied Biosystems, Foster City, CA, USA) on a StepOnePlus Real-Time PCR System (Applied Biosystems). Relative gene expression levels were calculated using the ΔΔCt method with glyceraldehyde-3-phosphate dehydrogenase (*GAPDH*) as the internal control. The qPCR cycling conditions comprised an initial denaturation step at 95 °C for 10 min, followed by 40 cycles of denaturation at 95 °C for 15 s, and annealing/extension at 60 °C for 1 min. The primers used for qPCR included sequences targeting C7, phosphatidylinositol-specific phospholipase C X domain containing 3 (*PLCXD3*), sodium leak channel non-selective protein (*NALCN*), laminin subunit alpha-2 (*LAMA2*), laminin subunit beta-1 (*LAMB1*), collagen type VI alpha 3 chain (*COL6A3*), and *GAPDH*, with specific forward and reverse primer sequences for *C7* (forward: 5′-GGCTGGCTGCGTGTTCTTGA-3′, reverse: 5′-CAGGAGTTGGAGACGAGCAT-3′), *PLCXD3* (forward: 5′-TTCCATGAATAAGATGGAGAAAGCTCATTG-3′, reverse: 5′-GTACCTTACCATTTACTGGGTACTTGCA-3′), *NALCN* (forward: 5′-ACTGACTTTGGTCCTGATGA-3′, reverse: 5′-ACTGAAGTGGAGGATAGTGC-3′), *LAMA2* (forward: 5′-ACGCTGAGAAACTTGAGGCCA-3′, reverse: 5′-TCAATATCTTTGAGGCAGCCGGA-3′), *LAMB1* (forward: 5′-CGCACCGCACGAGAGTTT-3′, reverse: 5′-CGCATTTACGTTCGCAAGAA-3′), *COL6A3* (forward: 5′-CCTAACCACATATGTTAGTGGAGGT-3′, reverse: 5′-GAATGTCTCGCTTGCTCTCTG-3′), and *GAPDH* (forward: 5′-GCTTCGCTCTCTGCTCCT-3′, reverse: 5′-CGCCCAATACGACCAAAT-3′).

### Small interfering RNA knockdown experiment in LX-2 cells

LX-2 cells, a known HSC cell line, were used to perform small interfering RNA (siRNA)-mediated knockdown of C7.^37^ Cells were maintained in DMEM supplemented with 10% FBS and 1% penicillin/streptomycin at 37 °C in a 5% CO_2_ incubator. Transfection was carried out using Lipofectamine™ RNAiMAX (Thermo Fisher Scientific, Waltham, MA, USA) according to the manufacturer’s protocol. Cells were transfected with siRNA targeting *C7* or non-targeting control siRNA at a final concentration of 30 nM. Cells were harvested on Day 3 for qPCR analysis to assess knockdown efficiency and evaluate the expression levels of *PLCXD3*, *NALCN*, *LAMA2*, *LAMB1*, and *COL6A3*.

### Statistical analysis

Clinical data were expressed as median (IQR) for continuous variables and number (percentage) for categorical variables. All statistical analyses were performed using StatFlex v7.0 (Artech Co., Ltd., Osaka, Japan), R software v4.3.0 (R Foundation for Statistical Computing), and GraphPad Prism v10 (GraphPad Software, San Diego, CA, USA).

Group comparisons were conducted using the Mann-Whitney *U* test or Kruskal-Wallis test for continuous variables, as appropriate, and the Chi-square test or Fisher’s exact test for categorical variables, as appropriate. Correlation analyses between clinical parameters and serum C7 levels were assessed using Spearman’s rank correlation coefficient. Multiple linear regression analyses were performed to identify independent predictors of fibrosis severity, and Cox proportional hazards regression was used for univariable and multivariable analyses of LRE risk.

Receiver operating characteristic (ROC) analysis was conducted to evaluate the diagnostic performance of serum C7, with the area under the ROC curve (AUROC) used as the primary metric. The optimal cut-off value was determined using Youden’s index. We used Kaplan-Meier survival analysis and log-rank testing to assess the cumulative incidence of LREs. All tests were two-sided, and a *p* value <0.05 was considered statistically significant.

## Results

### Identification of C7 as a candidate biomarker

To identify novel biomarkers associated with fibrosis severity and disease progression in MASLD, we conducted an integrated multi-omics analysis combining proteomic (Proteome-Phenome Atlas) and transcriptomic (GSE49541) datasets.[22], [23], [24] In the plasma proteomics dataset, C7 ranked the highest in terms of hazard ratio (HR) for predicting cirrhosis, showing the strongest association with disease risk (HR 24.99, 95% CI 19.88–31.42, *p* = 1.7×10^-167^; Fig. 2A). Other fibrosis-related proteins, including THBS2 and IGFBP7, also showed strong associations with disease progression as reported previously.[38], [39], [40], [41], [42]

**Fig. 2:**
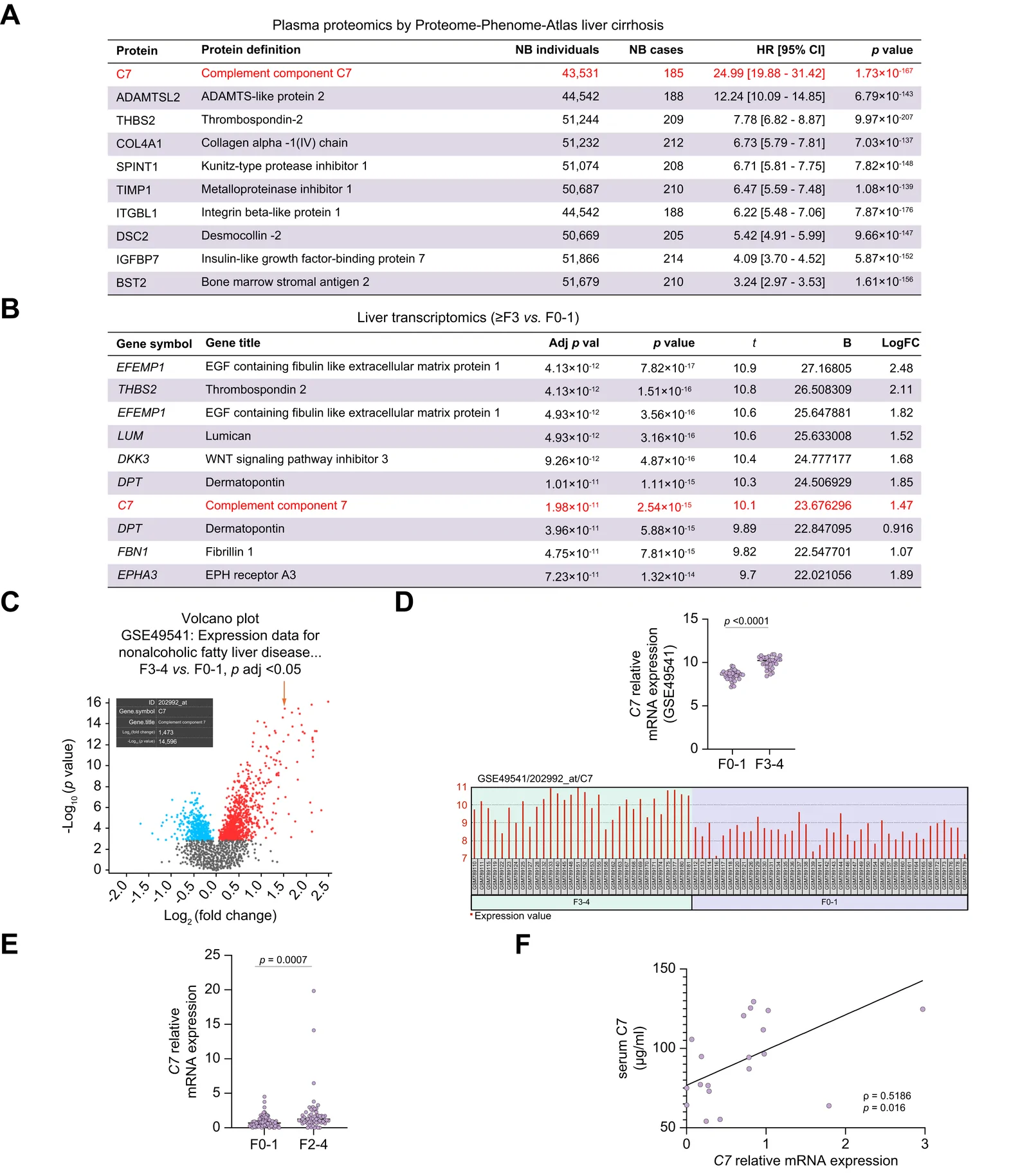
Multi-omics identification and validation of C7 as a fibrosis-associated biomarker in MASLD. (A) Table summarizing plasma proteomic analysis from the Proteome-Phenome Atlas, which reveals C7 as the plasma protein most strongly associated with fibrosis severity (HR 24.99, 95% CI 19.88–31.42, *p* = 1.73×10^-167^; as reported in the Proteome-Phenome Atlas). (B) Table showing the top 10 differentially expressed genes from GSE49541, which highlights *C7* as significantly upregulated in ≥F3 fibrosis cases (n = 32) *vs.* F0–1 cases (n = 40) (limma with Benjamini-Hochberg FDR correction; logFC = 1.47, adjusted *p* = 1.98×10^-11^). (C) Volcano plot depicting differentially expressed genes in GSE49541. *C7* (orange arrow) is a significantly upregulated gene in ≥F3 fibrosis cases (n = 32) *vs.* F0–1 cases (n = 40) (limma with Benjamini-Hochberg FDR correction; statistical significance is shown in the panel). (D) *C7* mRNA expression levels in GSE49541. The lower panel shows individual gene expression values for *C7* in ≥F3 (n = 32) and F0–1 (n = 40) cases. The upper panel presents a box plot summarizing the same data, which demonstrates significantly higher expression in ≥F3 fibrosis than in F0–1 (Mann-Whitney *U* test, *p* <0.0001). (E) qPCR validation of *C7* mRNA expression using in-house liver biopsy samples in F2–F4 fibrosis cases (n = 57) *vs.* F0–1 fibrosis cases (n = 103) (Mann-Whitney *U* test, *p* = 0.0007). (F) Correlation analysis between hepatic *C7* mRNA expression and serum C7 levels (n = 19) (Spearman’s rank correlation coefficient, ρ = 0.5186, *p* = 0.016). *C7*, complement component 7; F, fibrosis stage; FC, fold change; FDR, false discovery rate; HR, hazard ratio; MASLD, metabolic dysfunction-associated steatotic liver disease; qPCR, quantitative polymerase chain reaction.

Prompted by this striking proteomic finding, we next examined the expression of *C7* in liver tissue using the transcriptomic dataset. Differentially expressed genes between early (F0–1) and advanced (≥F3) fibrosis stages were first extracted. *C7* mRNA expression was significantly upregulated in advanced fibrosis (logFC = 1.47, adjusted *p* = 1.98×10^-11^; Fig. 2B–D). *C7* ranked as the seventh most upregulated gene in terms of fold change and was part of the highly significant fibrosis-related gene signature (Fig. 2B,C). The expression of *C7* was consistently elevated in ≥F3 fibrosis cases (Fig. 2D).

To validate the above findings, we performed qPCR analysis using in-house liver biopsy samples from patients with MASLD (n = 160; Fig. 2E). *C7* mRNA expression was significantly elevated in F2–F4 patients compared with F0–F1 patients (*p* = 0.0007; Fig. 2E), which confirmed its role in fibrosis progression. Moreover, hepatic *C7* mRNA levels correlated strongly with serum C7 levels (r = 0.5186, *p* = 0.016; Fig. 2F), suggesting a direct contribution of hepatic C7 production to circulating C7 levels. Our results indicated that C7 was strongly associated with fibrosis severity. Although C7 has long been recognized as a component of the terminal complement cascade, its role in MASLD remains unclear. Therefore, we conducted additional analyses to explore its potential involvement.

### Cohort characteristics

The baseline characteristics of patients with MASLD across different cohorts are summarized in Table S1–S3. In the Biopsy cohort, 430 patients with available serum samples were randomly assigned at a 3:1 ratio to the Main cohort (n = 323) and Sub cohort (n = 107) (Table S1). There were no significant differences in the clinicopathological characteristics between these groups apart from steatosis (*p* = 0.043). In the Main cohort, median patient age was 56 years, with 44% being male. Median BMI was 26.6 kg/m^2^, and the prevalence of DM, HT, and DL was 37%, 39%, and 63%, respectively. In the Sub cohort, median patient age was 57 years, with 42% being male. Median BMI was 27.1 kg/m^2^, and the prevalence of DM, HT, and DL was 39%, 48%, and 64%, respectively. Median serum C7 level was statistically comparable in the Main and Sub cohorts (124 *vs.* 125 μg/ml respectively, *p* = 0.247).

The Follow-up cohort (n = 348) consisted of patients with at least 1 year of longitudinal data. Median patient age was 56 years, with 41% being male (Table S2). Median BMI was 25.5 kg/m^2^, and the prevalence of DM, HT, and DL was 41%, 43%, and 62%, respectively. Median serum C7 level at baseline was 123.9 μg/ml. Over a median follow-up period of 7.1 (IQR 1.0–24.4) years, 22 (6.3%) patients experienced LREs including HCC in 12 (3.4%), ascites in 11 (3.2%), esophagogastric varices in six (1.7%), and hepatic encephalopathy in three (0.9%) patients. Notably, nine patients experienced multiple LREs concurrently, and 14 (4.0%) patients died during the observation period.

In the VCTE cohort (n = 120), median LSM was 6.1 kPa (Table S3). Median patient age was 59 years, with 55% being male. Median serum C7 level in this cohort was 118.1 μg/ml. At the time of MASLD diagnosis, 15 (12.5%) patients had a current or prior history of LREs, including HCC in 11 (9.2%), esophagogastric varices in five (4.2%), ascites in four (3.3%), and hepatic encephalopathy in two (1.7%) patients. Seven patients experienced multiple LREs.

### Association between serum C7 levels and fibrosis severity

Associations between serum C7 levels and fibrosis severity were consistently observed across multiple independent cohorts. In the Main cohort (*p* <0.0001) and the Sub cohort (*p* = 0.0486), serum C7 levels increased significantly with fibrosis stage, suggesting a potential role in fibrosis progression (Fig. 3A, left and middle panels). To identify the independent determinants of liver fibrosis severity, multiple regression analysis was performed in the Main cohort (Fig. 3A, right panel; Table S4). In the univariable analysis, age, male sex (inverse association), BMI, the presence of DM, alanine aminotransferase (ALT), and serum C7 levels were significantly associated with fibrosis stage. In the subsequent multivariable model, age (standardized β = 0.45, *p* <0.001), BMI (β = 0.28, *p* <0.001), ALT (β = 0.18, *p* <0.001), and serum C7 levels (β = 0.13, *p* = 0.007) remained independent predictors of fibrosis, whereas sex and DM lost significance.

**Fig. 3:**
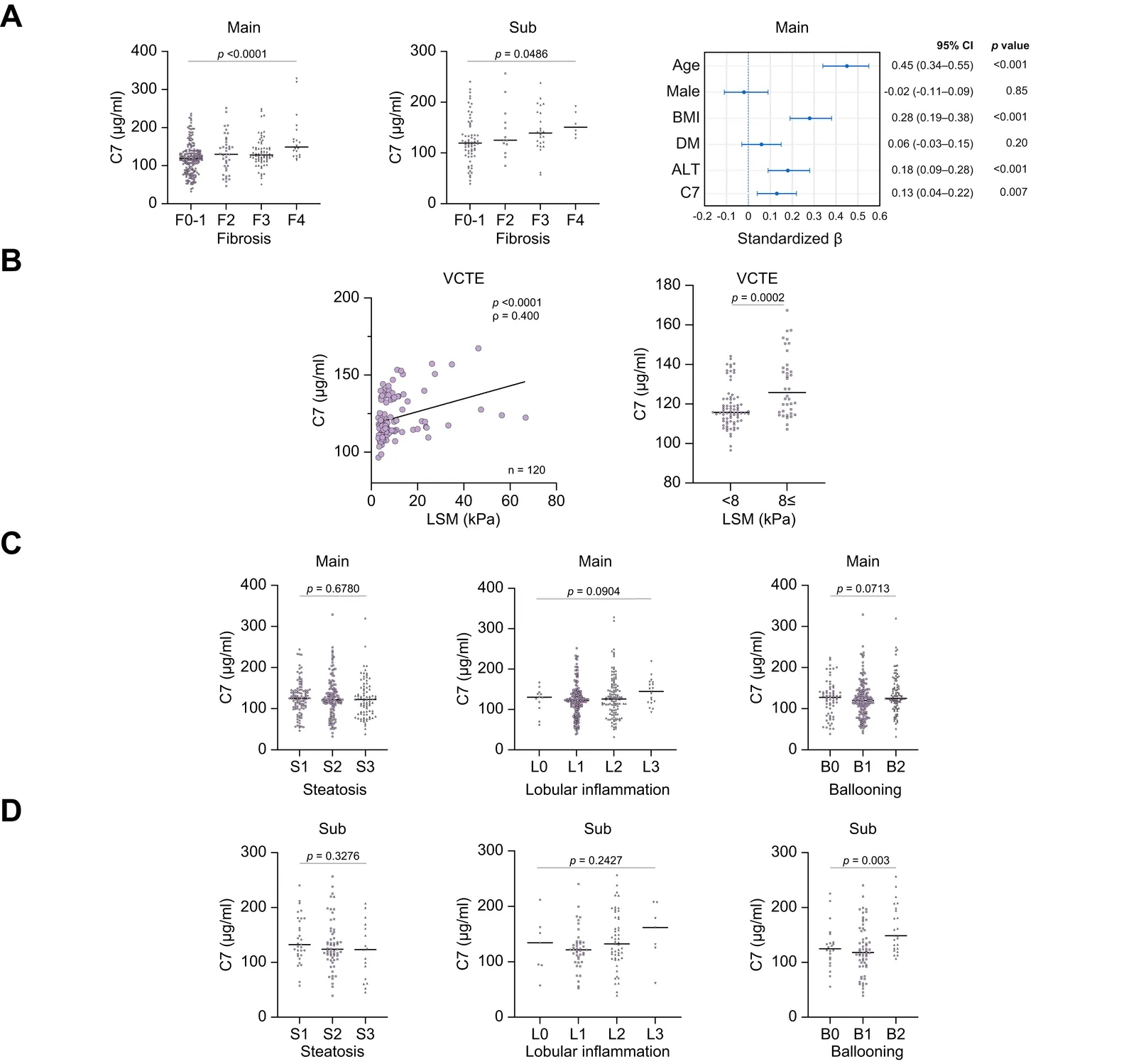
Association of serum C7 levels with fibrosis severity and histological features in MASLD. (A) Serum C7 levels stratified by fibrosis stage in the Main cohort (left, n = 323) and Sub cohort (middle, n = 107) (Kruskal-Wallis test; Main cohort, *p* <0.0001; Sub cohort, *p* = 0.0486). The right panel shows the multivariable linear regression analysis for factors associated with liver fibrosis in the Main cohort (multivariable linear regression analysis; exact *p* values are shown in the panel). (B) In the VCTE cohort (n = 120), correlation between serum C7 levels and LSM (left) (Spearman’s rank correlation coefficient, ρ = 0.400, *p* <0.001), and comparison of serum C7 levels between patients with LSM <8 kPa and those with LSM ≥8 kPa (right) (Mann-Whitney *U* test, *p* = 0.0002). (C) Serum C7 levels according to steatosis grade (S1–S3), lobular inflammation grade (L0–L3), and ballooning grade (B0–B2) in the Main cohort (Kruskal-Wallis test; steatosis, *p* = 0.6780; lobular inflammation, *p* = 0.0904; ballooning, *p* = 0.0713). (D) Corresponding analyses of steatosis, lobular inflammation, and ballooning in the Sub cohort (Kruskal-Wallis test; steatosis, *p* = 0.3276; lobular inflammation, *p* = 0.2427; ballooning, *p* = 0.003). B, ballooning; C7, complement component 7; L, lobular inflammation; LSM, liver stiffness measurement; MASLD, metabolic dysfunction-associated steatotic liver disease; S, steatosis; VCTE, vibration-controlled transient elastography.

This relationship was further validated in the VCTE cohort, where serum C7 levels demonstrated a significant positive correlation with LSM (r = 0.400, *p* <0.001), supporting its potential as a biomarker of fibrosis severity (Fig. 3B, left panel). Serum C7 levels were also significantly higher in patients with LSM ≥8 kPa than in those with LSM <8 kPa (*p* = 0.0002; Fig. 3B, right panel). In an additional stratified analysis, serum C7 levels increased significantly across clinically relevant LSM categories (<8, 8–12, 12–20, and ≥20 kPa; *p* = 0.003; Fig. S1C).

### Association between serum C7 levels and other histological features

Beyond its relationship with fibrosis severity, we examined the association between serum C7 levels and other histological features of MASLD. There was no significant correlation between serum C7 levels and steatosis in the Main cohort (*p* = 0.6780) or the Sub cohort (*p* = 0.3276) (Fig. 3C,D). Similarly, no remarkable association was found between serum C7 and lobular inflammation (*p* = 0.0904 in the Main cohort and *p* = 0.2427 in the Sub cohort; Fig. 3C,D). Conversely, serum C7 levels were significantly associated with hepatocellular ballooning in the Sub cohort (*p* = 0.003), with a non-significant trend in the Main cohort (*p* = 0.0713) (Fig. 3C,D). We also evaluated the relationship between serum C7 and NAS, but no significant association was observed in the Main cohort (*p* = 0.1530) or the Sub cohort (*p* = 0.6040) (Fig. S1B).

### Correlation between serum C7 levels and clinicopathological parameters

In the Main cohort, serum C7 levels were significantly correlated with several clinicopathological parameters (Table S5). Notably, we detected a moderate negative correlation with platelet count (r = -0.305, *p* <0.001), alongside positive correlations with serum AFP (r = 0.269, *p* <0.001), fibrosis stage (r = 0.232, *p* <0.001), and age (r = 0.203, *p* <0.001). We observed weaker but statistically significant associations with aspartate aminotransferase (AST), albumin, and gamma-glutamyl transferase levels. Conversely, no remarkable findings were found between C7 levels and other histological features, including steatosis, lobular inflammation, and NAS.

### Serum C7 levels and LREs

To evaluate the predictive value of serum C7 levels for LRE, we conducted ROC analysis in the Follow-up cohort to reveal serum C7 as a strong predictor of LREs, with an AUROC of 0.80. The optimal cut-off value for C7 was calculated as 140 μg/ml, yielding a sensitivity of 81.8% and a specificity of 72.1% (Fig. 4A). Kaplan-Meier survival analysis further revealed that patients with serum C7 levels ≥140 μg/ml had a significantly higher risk of developing LREs than those with serum C7 levels <140 μg/ml (log-rank *p* =0.00002; Fig. 4B). Moreover, patients who experienced LREs showed distinct clinical features, including older age, higher prevalence of DM, more advanced fibrosis, and significantly higher serum C7 levels, than those without LREs (Table S6).

**Fig. 4:**
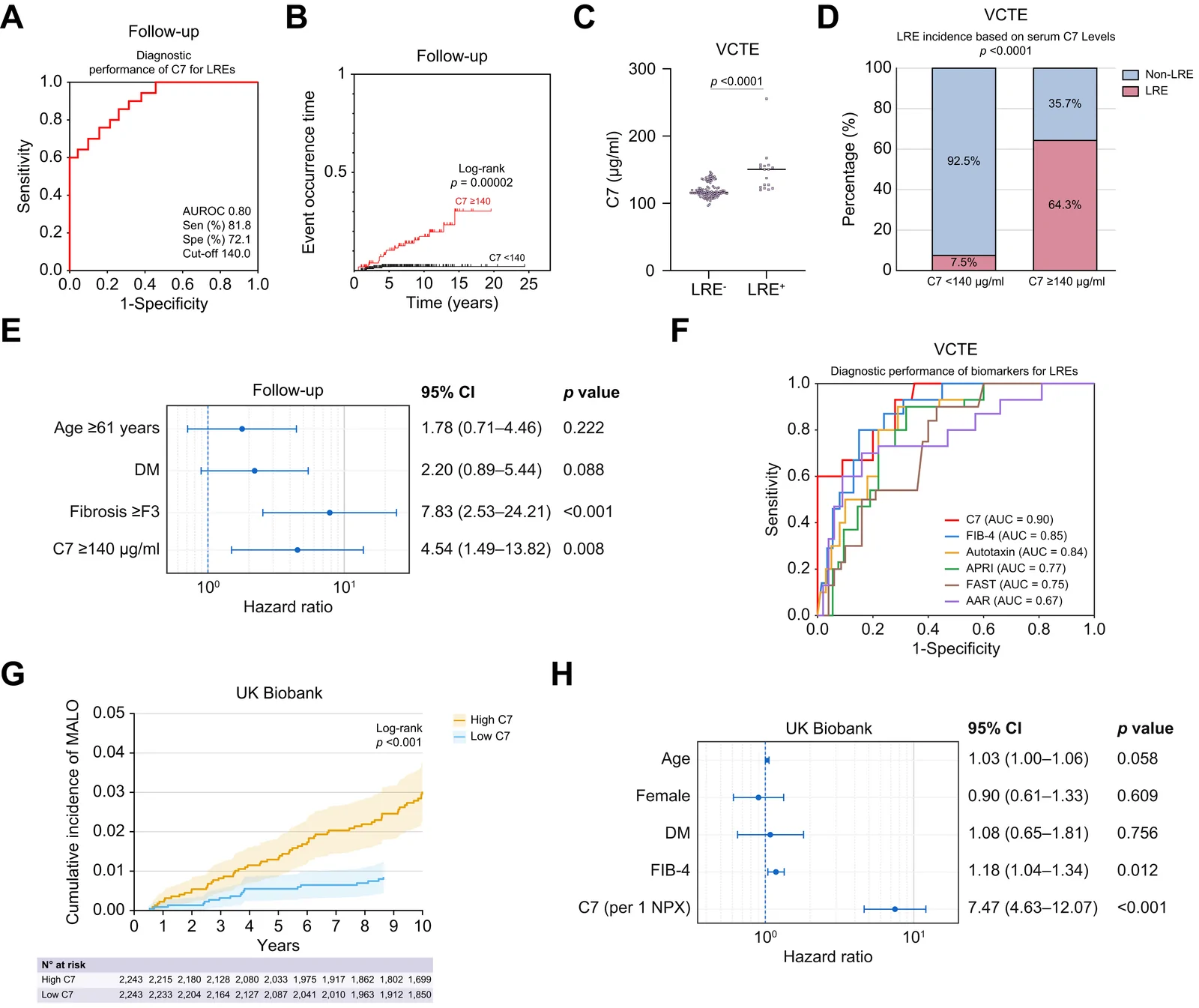
Association between serum C7 levels and liver-related outcomes, diagnostic performance, and external validation in MASLD. (A) Receiver operating characteristic (ROC) curve of serum C7 for predicting liver-related events (LREs) in the Follow-up cohort (n = 348). The area under the ROC curve (AUROC), sensitivity, specificity, and optimal cut-off value (140 μg/ml) are shown (ROC analysis; AUROC = 0.80; cut-off determined using Youden’s index). (B) Kaplan-Meier analysis comparing the cumulative incidence of LREs between patients with serum C7 levels ≥140 μg/ml and those with serum C7 levels <140 μg/ml in the Follow-up cohort (log-rank test, *p* =0.00002). (C) Serum C7 levels in patients with (LRE+) or without (LRE-) LREs in the VCTE cohort (n = 120) (Mann-Whitney *U* test, *p* <0.0001). (D) Stacked bar chart showing the incidence of LREs according to serum C7 levels (<140 or ≥140 μg/ml) in the VCTE cohort (Fisher’s exact test, *p* <0.0001). (E) Multivariable Cox proportional hazards model for LRE risk in the Follow-up cohort (Cox proportional hazards regression analysis; exact *p* values are shown in the panel). (F) ROC curves of six serum or non-invasive biomarkers (C7, FIB-4, autotaxin, APRI, FAST, and AAR) for identifying the presence of LREs in the VCTE cohort (ROC analysis; AUROC values are shown in the panel). (G) Cumulative incidence of MALOs according to serum C7 status in the UK Biobank cohort. Participants were dichotomized into low and high C7 groups based on the median C7 level (log-rank test, *p* <0.001). (H) Multivariable Cox proportional hazards model for MALOs in the UK Biobank cohort (Cox proportional hazards regression analysis; exact *p* values are shown in the panel). AAR, aspartate aminotransferase-to-alanine aminotransferase ratio; APRI, aspartate aminotransferase-to-platelet ratio index; AUROC, area under the receiver operating characteristic curve; C7, complement component 7; DM, diabetes mellitus; FAST, FibroScan-AST; FIB-4, fibrosis-4 index; LRE, liver-related event; MALOs, major adverse liver outcomes; MASLD, metabolic dysfunction-associated steatotic liver disease; VCTE, vibration-controlled transient elastography.

To validate these findings, serum C7 levels were examined in the VCTE cohort, in which patients experiencing LREs displayed significantly higher serum C7 levels than those who did not (*p* <0.0001; Fig. 4C). Stratification of patients based on serum C7 levels showed a markedly higher incidence of LREs for serum C7 levels ≥140 μg/ml (64.3%) *vs.* <140 μg/ml (7.5%) (*p* <0.0001; Fig. 4D).

Multivariable Cox proportional hazards analysis in the Follow-up cohort confirmed that both advanced fibrosis (≥F3) and elevated serum C7 levels (≥140 μg/ml) were independent predictors of LRE risk (Fig. 4E and Table S7). The HR was 7.83 (95% CI 2.53–24.21, *p* <0.001) for advanced fibrosis and 4.54 (95% CI 1.49–13.82, *p* = 0.008) for elevated serum C7 levels. Neither age nor DM remained significant predictors of LREs after multivariable adjustment. These findings highlighted serum C7 as a robust and independent predictor of LREs in patients with MASLD, supporting a potential clinical utility in risk stratification.

### Serum biomarkers associated with the presence of LREs

In the VCTE cohort, we conducted ROC analysis to compare the diagnostic performance of six serum or non-invasive biomarkers—C7, FIB-4 index, autotaxin, AST-to-platelet ratio index (APRI), AST-to-ALT ratio (AAR), and FAST—for detecting the presence of LREs. C7 showed the highest diagnostic ability (AUROC = 0.90), followed by FIB-4 index, autotaxin, APRI, FAST, and AAR (AUROC 0.85, 0.84, 0.77, 0.75, and 0.67, respectively; Fig. 4F).

### External validation in the UK Biobank cohort

To externally validate the clinical relevance of C7, we analyzed 4,486 participants with MASLD from the UK Biobank cohort. MALOs occurred more frequently in the high C7 group than in the low C7 group (89 *vs*. 32 events), and cumulative incidence curves showed a significant separation between the two groups (log-rank, *p* <0.001; Fig. 4G). The 5-year cumulative incidence was 1.29% in the high C7 group and 0.55% in the low C7 group, increasing to 3.02% and 0.85% at 10 years, respectively. In multivariable Cox regression analysis, C7 was independently associated with MALOs after adjustment for FIB-4 (HR 7.47, 95% CI 4.63–12.07, *p* <0.001; Fig. 4H and Table S13). These findings support the external relevance of C7 across multiple MASLD cohorts.

### Spatial transcriptomics and ISH analysis of *C7* expression

As circulating C7 levels strongly correlated with hepatic *C7* mRNA expression, we sought to identify the C7-producing cells in the fibrotic MASLD liver. Spatial transcriptomic analysis revealed a heterogeneous pattern of *C7* expression, with notably higher levels concentrated in fibrotic regions of F3 patients compared with F0 patients (Fig. 5A).

**Fig. 5:**
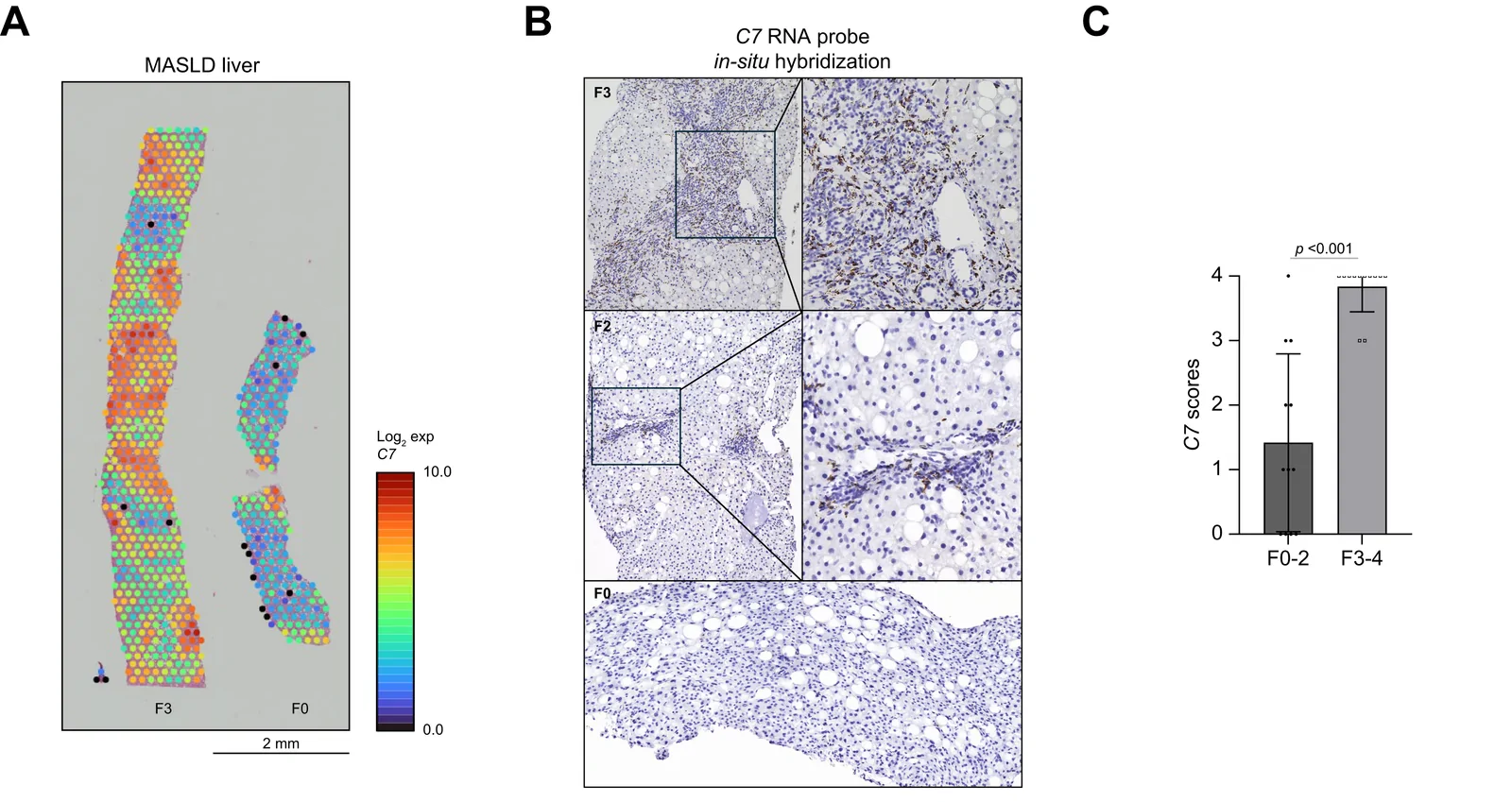
Spatial and histological localization of *C7* mRNA expression in MASLD liver tissue. (A) Spatial transcriptomic map showing the distribution of *C7* expression (log2 scale) in liver tissue from MASLD patients with advanced fibrosis (≥F3) and no fibrosis (F0). (B) Representative images of ISH for *C7* mRNA in liver tissue from MASLD patients with fibrosis stages F3, F2, and F0. (C) Semi-quantitative ISH scores of *C7* expression based on a five-grade scoring system compared between patients with mild (F0–2) and advanced (≥F3) fibrosis (n = 12 each). Bars represent the median with IQR. Statistical significance was determined using the Mann-Whitney *U* test (*p* <0.001). *C7*, complement component 7; F, fibrosis stage; ISH, *in situ* hybridization; MASLD, metabolic dysfunction-associated steatotic liver disease.

To validate these findings at the histological level, we performed RNA ISH using a probe targeting *C7* mRNA, which demonstrated prominent *C7* expression in fibrotic areas of liver tissue from patients with MASLD and advanced fibrosis (≥F3). *C7* mRNA signals were also observed in perisinusoidal regions where HSCs are known to reside, suggesting that *C7* was primarily expressed by non-parenchymal cells rather than hepatocytes. Quantitative scoring of ISH signals using a five-grade scale revealed significantly higher *C7* expression in patients with advanced fibrosis than in those with minimal fibrosis (F0–2) (*p* <0.001; Fig. 5C).

To further support the protein-level localization of C7, double immunofluorescence staining for C7 and α-SMA was performed in F4 liver tissue. C7 protein signals were observed within fibrotic tissue compartments, particularly in proximity to α-SMA-positive fibrogenic areas (Fig. S1D).

### snRNA-seq of C7 expression

We next aimed at elucidating the specific cell populations responsible for *C7* expression in MASLD-related cirrhosis using a publicly available snRNA-seq dataset (GSE174748).^26^ Unsupervised clustering using Uniform Manifold Approximation and Projection (UMAP) identified distinct liver cell populations, including hepatocytes, endothelial cells, HSCs, immune cells, and cholangiocytes (Fig. 6A). Clustering across different conditions revealed these populations to be comparable between healthy and MASLD cirrhosis samples (Fig. 6B). To explore cell type-specific changes in *C7* expression, violin plots were generated for different liver cell types under healthy and MASLD conditions (Fig. 6C). Among these, significant differences in *C7* expression were observed in HSCs (*p* = 0.01) and endothelial cells (*p* = 1.1×10^-5^), with none in hepatocytes (*p* = 0.85). Specifically, *C7* expression was significantly upregulated in HSCs of MASLD cirrhosis but was downregulated in endothelial cells (Fig. 6D). HSCs were therefore suspected to be a key source of increased C7 production in MASLD, whereas endothelial cells might contribute to its clearance or regulation.

**Fig. 6:**
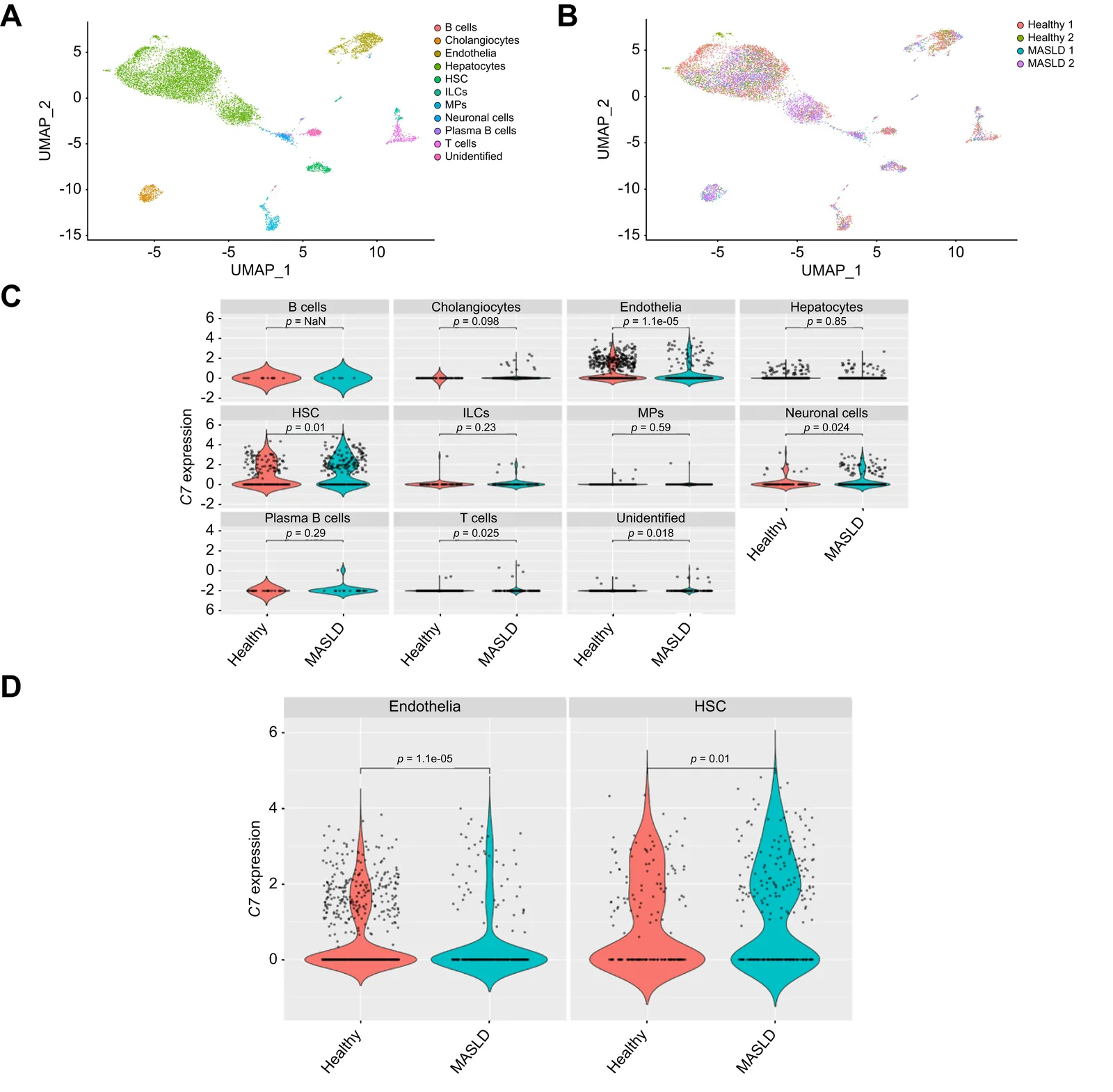
snRNA-seq analysis of *C7* expression across liver cell types in healthy and MASLD cirrhosis conditions. (A) UMAP plot showing clustering of liver cell populations annotated by cell type. (B) UMAP plot displaying cell clusters colored by sample group (healthy: n = 2, MASLD cirrhosis, n = 2). (C) Violin plots of *C7* expression across multiple liver cell types under healthy and MASLD cirrhosis conditions (Wilcoxon rank-sum test; exact *p* values are shown in the panel). (D) Violin plots highlighting *C7* expression differences in endothelial cells and HSCs between healthy and MASLD cirrhosis conditions (Wilcoxon rank-sum test; endothelial cells, *p* = 1.1×10^-5^; HSCs, *p* = 0.01). *C7*, complement component 7; Endothelia, endothelial cells; HSC, hepatic stellate cell; ILC, innate lymphoid cell; MASLD, metabolic dysfunction-associated steatotic liver disease; MP, mononuclear phagocyte; snRNA-seq, single-nucleus RNA sequencing; UMAP, Uniform Manifold Approximation and Projection.

### Functional implications of C7 in HSCs

We conducted correlation and pathway enrichment analyses to explore the molecular interactions of C7 in HSCs under fibrotic MASLD conditions. Pearson correlation analysis identified the top 50 genes positively associated with *C7* expression in HSCs, among which *PLCXD3*, *NALCN*, *LAMA2*, *LAMB1*, and *COL6A3* showed strong relationships (Fig. 7A). Functional enrichment analysis revealed that these co-expressed genes were enriched in fibrosis-related pathways, including the PI3K-Akt signaling pathway and ECM organization (Fig. 7B).

**Fig. 7:**
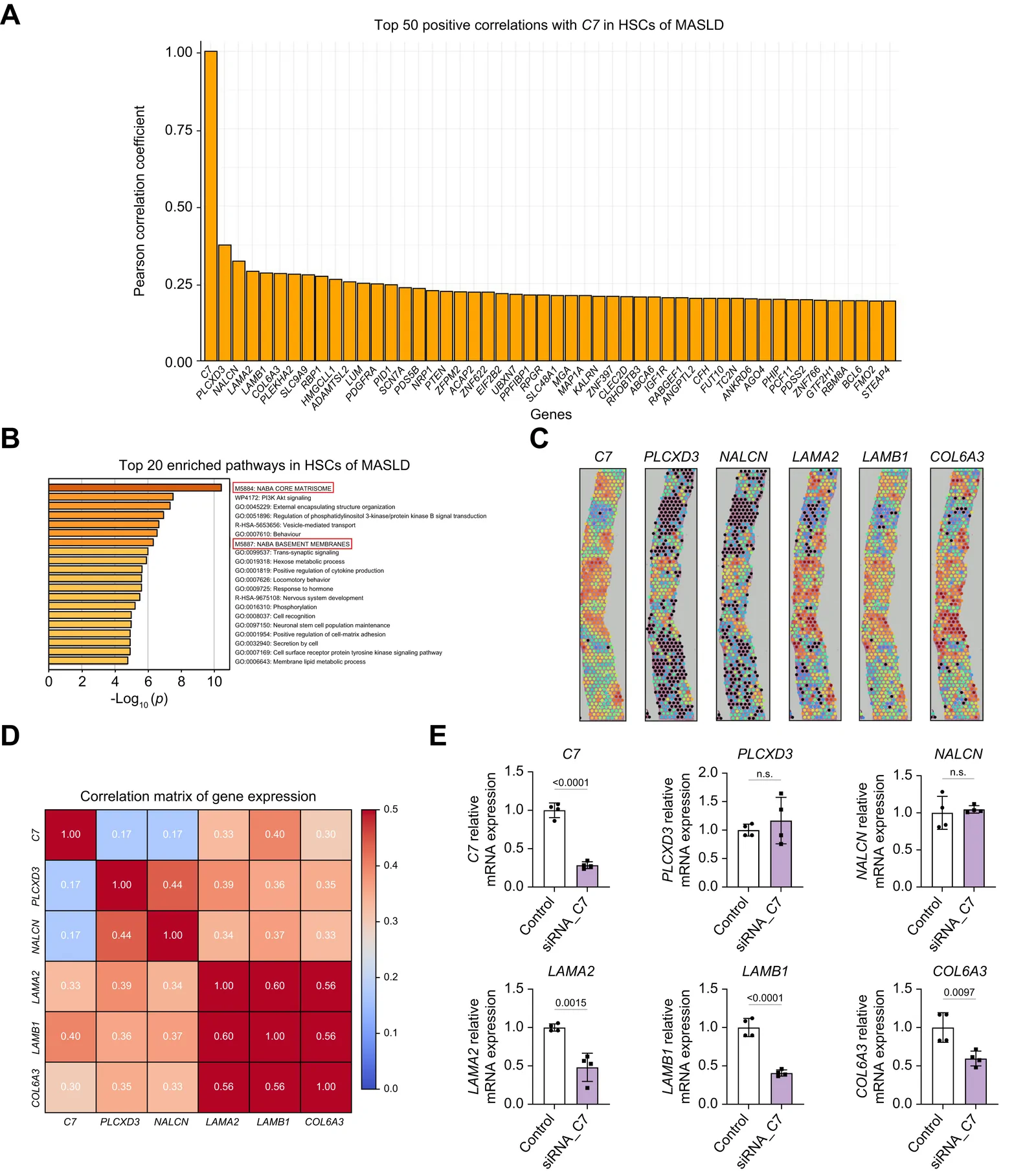
Gene network and functional impact of *C7* expression in HSCs under MASLD cirrhosis conditions. (A) Top 50 genes positively correlated with *C7* expression in HSCs from MASLD cirrhotic liver tissue based on single-nucleus transcriptomic analysis (GSE174748). Pearson correlation coefficients are shown in the panel (Pearson correlation analysis). (B) Top 20 enriched pathways among *C7*-correlated genes in HSCs under MASLD cirrhosis conditions identified by gene ontology and pathway enrichment analysis (pathway enrichment analysis; adjusted *p* values are shown in the panel). (C) Spatial transcriptomic visualization of *C7* and selected correlated genes (*PLCXD3*, *NALCN*, *LAMA2*, *LAMB1*, and *COL6A3*) in liver sections from patients with MASLD (F0 *vs*. F3). (D) Correlation matrix depicting the relationship between *C7* and the expression of selected ECM-related genes in HSCs (Pearson correlation analysis). (E) qPCR analysis showing mRNA expression levels of *C7* and selected ECM-related genes in LX-2 cells after siRNA-mediated knockdown of *C7*. Data are presented as mean ± SEM (Mann-Whitney *U* test; exact *p* values are shown in the panel). *C7*, complement component 7; *COL6A3*, collagen type VI alpha 3 chain; ECM, extracellular matrix; F, fibrosis stage; HSC, hepatic stellate cell; *LAMA2*, laminin subunit alpha-2; *LAMB1*, laminin subunit beta-1; MASLD, metabolic dysfunction-associated steatotic liver disease; *NALCN*, sodium leak channel non-selective protein; ns, not significant; *PLCXD3*, phosphatidylinositol-specific phospholipase C X domain containing 3; qPCR, quantitative polymerase chain reaction; siRNA, small interfering RNA.

To further explore the microenvironmental context of *C7*-enriched regions, we performed spatial transcriptomic module-score analyses using predefined signatures representing ECM/HSC-related fibrogenic activity, progenitor/ductular reaction, and hypoxia-related stress. These signatures were constructed from representative genes corresponding to each biological program, with the detailed gene sets provided in the Supplementary Methods. Spatial mapping demonstrated that *C7*-enriched regions closely overlapped with areas having high ECM/HSC-related fibrogenic scores and were accompanied by regions exhibiting a progenitor/ductular reaction, whereas the hypoxia-related signal was more modestly distributed (Fig. S3A). Consistently, *C7* expression showed the strongest correlation with the ECM/HSC-related fibrogenic score (Spearman ρ = 0.819, *p* <0.001), followed by the progenitor/ductular reaction score (ρ = 0.708, *p* <0.001), whereas a weaker but significant association was observed with the hypoxia-related score (ρ = 0.341, *p* <0.001) (Fig. S3B). Spatial overlap analysis further showed that many *C7*-high spots overlapped with progenitor-high spots within the fibrotic tissue architecture (Fig. S3C). These findings indicate that *C7*-enriched regions are spatially associated primarily with an ECM/HSC-rich fibrotic niche and secondarily with progenitor/ductular reaction programs and hypoxia-related microenvironmental stress.

Spatial transcriptomic analysis further confirmed the colocalization of *C7* with *PLCXD3*, *NALCN*, *LAMA2*, *LAMB1*, and *COL6A3* within fibrotic liver regions, supporting their functional relevance in fibrosis progression (Fig. 7C). A gene correlation matrix demonstrated moderate correlations between *C7* and ECM-related genes, particularly *LAMA2*, *LAMB1*, and *COL6A3* (Fig. 7D). To experimentally validate the direct contribution of C7 to the fibrogenic pathway, we performed siRNA-mediated knockdown of *C7* in LX-2 cells, a known human HSC line. This knockdown significantly reduced the expression of *LAMA2*, *LAMB1*, and *COL6A3*, whereas *PLCXD3* and *NALCN* expression remained unchanged (Fig. 7E). Thus, C7 likely regulates ECM remodeling in HSCs, potentially contributing to fibrosis progression in MASLD.

### Functional implications of C7 in endothelial cells

Lastly, we performed correlation and pathway enrichment analyses to investigate the role of *C7* in endothelial cells under MASLD conditions. Pearson correlation analysis identified the top 50 genes positively associated with *C7* expression in endothelial cells, which included *FGF3*, *FSTL1*, *APLNR*, and *VEGFA* (Fig. S2A). Notable associations with *C7* were seen for genes involved in angiogenesis and endothelial function. Pathway enrichment analysis also revealed significant enrichment in vascular endothelial growth factor (VEGF) signaling, enzyme-linked receptor signaling, and cell junction organization pathways (Fig. S2B). These findings suggest that *C7* influenced endothelial function in MASLD through pathways related to vascular remodeling and cellular signaling.

### Association of serum C7 with complement activation intermediates

To further examine whether elevated C7 is linked to broader complement activation, we measured the serum complement activation intermediates C5a and soluble C5b-9. Serum C5a showed a modest but significant positive correlation with serum C7 (Spearman ρ = 0.22, *p* = 0.046), whereas soluble C5b-9 showed a positive trend with serum C7 that did not reach statistical significance (Spearman ρ = 0.20, *p* = 0.071) (Fig. S4). These findings indicate that elevated C7 may be associated with complement activation in MASLD.

## Discussion

### Main findings

In this study, we identified complement C7 as a novel biomarker associated with fibrosis progression and LREs in MASLD using a comprehensive multi-omics approach. C7 initially emerged as the top-ranking protein in plasma proteomics based on HR for predicting cirrhosis (Fig. 2A), prompting further investigation into its biological significance. Indeed, *C7* expression was elevated in fibrotic liver tissue. Spatial and single-nucleus transcriptomic analyses localized *C7* expression predominantly to HSCs, with enrichment in fibrotic regions. Serum C7 levels were consistently associated with fibrosis stage and LSM across independent cohorts. Importantly, high serum C7 levels (≥140 μg/ml) were independently associated with increased LRE risk, even after adjusting for fibrosis severity. This prognostic relevance was further supported by external validation in the UK Biobank cohort, in which higher plasma C7 was independently associated with MALOs after adjustment for FIB-4 and clinical covariates. C7 also showed higher diagnostic ability for LREs than conventional indices. *In vitro* siRNA-mediated knockdown of *C7* in LX-2 cells led to downregulation of key ECM-related genes, thereby highlighting its functional role in fibrogenesis. Taken together, these findings support the potential utility of serum C7 as a non-invasive biomarker for risk stratification and monitoring in patients with MASLD.

### Context with published literature

C7 is a terminal component of the complement cascade that is crucial for membrane attack complex formation and classical immune defense.^43^ However, complement proteins, including C7, are now recognized to participate in non-cytolytic processes such as inflammation, tissue remodeling, and fibrosis in chronic diseases.^44^ The liver is a major site of complement synthesis, with hepatocytes being their primary producers.^45^ Recent evidence has suggested the contribution of non-parenchymal cells, such as HSCs, liver sinusoidal endothelial cells, and Kupffer cells, to local complement regulation, particularly in disease states.^46^^,^^47^ Nonetheless, the specific role of C7 in chronic liver diseases is not fully understood.

Our data expand the current body of knowledge by revealing increased *C7* expression in activated HSCs in MASLD. This cell type specificity was consistently observed in spatial transcriptomic and snRNA-seq analyses. As HSCs are central to fibrogenesis via ECM production, elevated *C7* expression in these cells suggests its involvement in the fibrotic remodeling process. Accordingly, siRNA knockdown of *C7* in LX-2 cells suppressed key ECM genes, such as *COL6A3* and *LAMB1*, suggesting that *C7* modulates fibrogenic activity. However, *C7* expression was significantly downregulated in endothelial cells under MASLD conditions. Given that endothelial cell dysfunction contributes to capillarization, inflammation, and disease progression, the loss of *C7* may reflect altered immunoregulatory or structural roles of the endothelium in fibrotic settings.^48^ Endothelial-derived complement components have been shown to affect leukocyte adhesion and vascular remodeling.^49^ Thus, *C7* downregulation might represent a compensatory or pathogenic adaptation in this context.

Our findings both corroborate and substantially extend previous reports on C7 function. Hou *et al.*^44^ identified hepatic *C7* as a gene associated with advanced fibrosis in NAFLD, whereas Magdy *et al.*^46^ reported its transcriptional upregulation and hypomethylation in liver tissues from patients with advanced MASLD. The present study extends these observations by showing that C7 is not only a fibrosis-associated marker but also a prognostic biomarker for LRE development. Serum C7 levels predicted LREs independently of fibrosis stage in multivariable analyses, suggesting that C7 reflects pathological processes beyond the extent of structural liver damage. In addition, our data link C7 to complement activation and HSC-related fibrogenic function across multiple analytical layers, supporting its role as part of a broader disease axis relevant to clinical progression.

Consistent with this interpretation, spatial module-score analyses further showed that *C7*-enriched regions were primarily associated with an ECM/HSC-rich fibrotic niche and secondarily with progenitor/ductular reaction programs and hypoxia-related microenvironmental stress. These findings provide additional spatial context suggesting that *C7* is embedded within a tissue-remodeling microenvironment in fibrotic MASLD liver.

The behavior of serum C7 may differ across liver disease etiologies. In primary biliary cholangitis, serum C7 elevation and C7-containing proteomic signatures have been reported,^50^^,^^51^ whereas direct evidence for serum C7 as a fibrosis biomarker in autoimmune hepatitis or primary sclerosing cholangitis remains limited. Therefore, further studies are warranted to determine whether serum C7 reflects fibrosis, cholestatic injury, immune-mediated injury, or broader disease-specific processes in non-MASLD liver diseases.

Mechanistically, Seol *et al.*^52^ provided evidence of high C7 expression in tumor-forming cells in the liver to promote the transcription of key stemness-related genes, including *OCT4*, *Nanog*, *SOX2*, and *c-Myc*, in an LSF-1-dependent manner. This finding highlighted a broader biological role for C7 in hepatocarcinogenesis, and may partly explain the elevated risk of HCC and other LREs in patients with high serum C7 levels in our study. Zhang *et al.*^53^ identified *C7* as a component of a hypoxia- and stemness-related gene signature that estimated prognosis and immune checkpoint responses in HCC. In their hypoxic model, *C7* expression was downregulated under hypoxic stress, thereby linking it to the tumor microenvironment and immunomodulation.

### Strengths and limitations

The major strength of this study lies in its integrative, multi-omics design that incorporates transcriptomics, proteomics, spatial and single-cell analyses, as well as *in vitro* functional validation. These findings were consistently reproduced across biopsy-proven and VCTE-based clinical cohorts and were further supported by external validation in the UK Biobank cohort. The identification of C7 as an independent predictor of LREs reinforces its potential utility as a non-invasive biomarker for clinical risk stratification and decision-making. Notably, ROC analysis demonstrated that serum C7 levels had the highest diagnostic accuracy (AUROC = 0.90) for distinguishing patients with LREs, outperforming established biomarkers including the FIB-4 index, autotaxin, APRI, FAST, and AAR.

However, several limitations should be acknowledged. First, the follow-up cohort spanned a long period, during which changes in diagnostic criteria, clinical management, and sample handling may have introduced heterogeneity and potential batch-related effects. Second, although the external validation in the UK Biobank cohort supports the broader relevance of our findings, differences in cohort structure, endpoint definitions, and available variables across datasets should be considered in terms of generalizability. Third, although functional evidence from LX-2 cells supports the role of *C7* in fibrogenesis, *in vivo* mechanistic studies are warranted to establish causality. Liver fibrosis models using *C7*-deficient mice, such as *C7* knockout models, or neutralizing antibodies against C7 will help elucidate its pathogenic role *in vivo*. Lastly, although our data highlight HSCs as the dominant source of *C7*, the molecular mechanisms underlying the upregulation of *C7* mRNA in HSCs remain unclear. To date, no specific transcription factor has been reported to directly modulate *C7* expression through promoter binding. Further study is needed to identify the upstream regulators of *C7* transcription in the context of liver fibrosis.

### Future implications

Serum C7 serves as a promising non-invasive biomarker for stratifying fibrosis severity and predicting clinical outcomes in MASLD. Beyond diagnostics, the cell type-specific expression pattern of *C7* suggests a role as a therapeutic target. Mechanistic studies of C7 signaling in HSCs and its interaction with inflammatory, angiogenic, and oncogenic pathways are needed. Validation in prospective cohorts is also essential to establish its clinical utility in treatment strategies and long-term monitoring of patients with MASLD.

## Authors’ contributions

Study conception and design: SK, TK, TI. Collection of clinical data: TI, TK, TO, SW, HK, YY, SI, TY. Conducted experiments and generated analytical data: SK, TI. Data analysis and interpretation of results: SK, TI, TK. Manuscript drafting: SK, TI, TK. Provided technical and bioinformatics support: MT, SJ, SC. Contributed to histological assessment and clinical interpretation: TN, MI, T Uehara. Had authorized access to the UK Biobank data and performed the UK Biobank analysis: TM. Scientific supervision and critical revision of the manuscript: NF, T Umemura, BS, NT.

## Data availability

Publicly available datasets used in this study are available from the Gene Expression Omnibus under accession numbers GSE49541 and GSE174748 and from the Proteome-Phenome Atlas. UK Biobank data were accessed under application number 930515 and are available to approved researchers through UK Biobank. Other data generated or analyzed in this study are not publicly available due to ethical and privacy restrictions but may be available from the corresponding author upon reasonable request and with appropriate institutional approval.

## Abbreviations

AAR, aspartate aminotransferase-to-alanine aminotransferase ratio; AFP, alpha-fetoprotein; ALT, alanine aminotransferase; APRI, aspartate aminotransferase-to-platelet ratio index; AST, aspartate aminotransferase; AUROC, area under the receiver operating characteristic curve; C7, complement component 7; cDNA, complementary DNA; *COL6A3*, collagen type VI alpha 3 chain; DL, dyslipidemia; DM, diabetes mellitus; ECM, extracellular matrix; F, fibrosis stage; FAST, FibroScan-AST; FDR, false discovery rate; *FIB-4*, fibrosis-4 index; *GAPDH*, glyceraldehyde-3-phosphate dehydrogenase; GEO, Gene Expression Omnibus; GO, Gene Ontology; HCC, hepatocellular carcinoma; HR, hazard ratio; HSC, hepatic stellate cell; HT, hypertension; ILC, innate lymphoid cell; ISH, *in situ* hybridization; KEGG, Kyoto Encyclopedia of Genes and Genomes; *LAMB1*, laminin subunit beta-1; *LAMA2*, laminin subunit alpha-2; LRE, liver-related event; LSM, liver stiffness measurement; MALOs, major adverse liver outcomes; MASLD, metabolic dysfunction-associated steatotic liver disease; MP, mononuclear phagocyte; NAFLD, non-alcoholic fatty liver disease; NAS, NAFLD activity score; *NALCN*, sodium leak channel non-selective protein; NASH, non-alcoholic steatohepatitis; *PLCXD3*, phosphatidylinositol-specific phospholipase C X domain containing 3; qPCR, quantitative PCR; ROC, receiver operating characteristic; snRNA-seq, single-nucleus RNA sequencing; siRNA, small interfering RNA; UMAP, Uniform Manifold Approximation and Projection; VCTE, vibration-controlled transient elastography.

## Financial support

This work was supported by the 10.13039/100009619Japan Agency for Medical Research and Development (AMED; JP25fk0210125 and JP26fk0210190 to TK and NT; JP256f0137007 to TK and SW) and the 10.13039/501100001691Japan Society for the Promotion of Science (JSPS KAKENHI: JP24K11087 to TK, JP25K19323 to TO, JP25K20541 to SW, and 24K18908 to TM), and the Indian Council of Medical Research (ICMR), Government of India (EMDR/IG/13/2023-0384 to SJ). This study was supported by services provided by the National Institutes of Health center P30 DK120515.

## Conflicts of interest

BS has been consulting for Boehringer Ingelheim Pharma and Mabwell Therapeutics (prior 24 months). BS’s institution UC San Diego (CA, USA) has received research support from Axial Biotherapeutics, Apollo Therapeutics, ChromoLogic, Intercept Pharmaceuticals, and Prodigy Biotech (prior 24 months). BS is the founder of Nterica Bio. UC San Diego has filed several patents with BS designated as an inventor. All the other authors declare no competing interests related to this manuscript.

Please refer to the accompanying ICMJE disclosure forms for further details.
