# Supplementary material for: Integrative multi-omics analysis identifies stellate cell-derived complement component 7 as a predictor of fibrosis and liver-related events in MASLD
Source: JHEP Rep. 2026 Jul 22;8(11):101965. doi: 10.1016/j.jhepr.2026.101965 (PMC13651563; doi:10.1016/j.jhepr.2026.101965)
Supplement: Supplementary file 1 [file mmc1.pdf]

Integrative multi-omics analysis identifies stellate cell-derived complement component 7 as a predictor of fibrosis and liver-related events in MASLD

Shohei Kondo, Takefumi Kimura, Takanobu Iwadare, Mohammad Taufeeq, Takao Miwa, Naoyuki Fujimori, Tomoyuki Nakajima, Shun-ichi Wakabayashi, Taiki Okumura, Hiroyuki Kobayashi, Yuki Yamashita, Satsuki Isogai, Tomoo Yamazaki, Mai Iwaya, Takeshi Uehara, Takeji Umemura, Saket Choudhary, Bernd Schnabl, Shanu Jain, Naoki Tanaka

Table of contents

Supplementary methods..2

Supplementary figure legends..4

Supplementary figures..7

Supplementary tables..11

Supplementary methods

UK Biobank Cohort

External validation was conducted using the UK Biobank, a large prospective population-based cohort. Among participants with available serum C7 measurements, those with missing alcohol intake data, alcohol intake above the MASLD threshold, baseline liver disease, no observational period, or previous major adverse liver outcomes were excluded. After assessment for MASLD and exclusion of participants who did not meet the MASLD criteria, 4,486 participants were included in the final analysis. Plasma C7 levels were measured using the Olink Explore platform and are expressed as normalized protein expression (NPX) values on a log2 scale. A flow diagram of participant selection is shown in Supplementary Figure 1A, and baseline characteristics of participants with MASLD according to C7 status are summarized in Supplementary Table 8.

Definition of MASLD in the UK Biobank Cohort

Hepatic steatosis was defined using the fatty liver index (FLI) ≥60. Cardiometabolic risk factors were assessed on the basis of body mass index and waist circumference, glycemic status, triglycerides, HDL-cholesterol, and blood pressure or relevant medication use. Alcohol intake (g/day) was estimated from self-reported beverage-specific consumption using standard ethanol conversion factors. MASLD was defined as hepatic steatosis together with at least one cardiometabolic risk factor and alcohol intake within the specified threshold. ICD-10 codes used to identify previous chronic liver diseases are listed in Supplementary Table 9, and beverage-specific ethanol conversion factors are provided in Supplementary Table 11.

Definition of Major Adverse Liver Outcomes

Major adverse liver outcomes (MALO) were defined using registry-based diagnoses of ascites, cirrhosis, varices, hepatocellular carcinoma, liver failure, liver transplantation, and other major liver outcomes identified by ICD-10 codes. Detailed diagnostic codes are listed in Supplementary Table 10.

Missing Data Handling

Among participants assessed for MASLD, missing values in covariates other than

C7 were handled using multiple imputation by chained equations. Details of the missing data distribution and imputation procedure are provided in Supplementary Table 12.

Double Immunofluorescence Staining

Double immunofluorescence staining was performed on F4 liver tissue to evaluate the protein-level localization of C7 in relation to activated hepatic stellate cell/myofibroblast areas. The following primary antibodies were used: anti-C7 antibody (rabbit polyclonal, HPA001465; Sigma-Aldrich/Merck KGaA, Darmstadt, Germany) and anti-α-SMA antibody (mouse monoclonal, clone 1A4, M0851; Dako/Agilent Technologies, Santa Clara, CA, USA). Nuclei were counterstained with DAPI. Representative images are shown in Supplementary Figure 1D.

Spatial Module-Score Analysis

To further characterize the spatial microenvironment associated with C7-enriched regions, spatial module-score analyses were performed using predefined gene sets representing ECM/HSC-related fibrogenic programs, hypoxia-related signatures, and progenitor/ductular reaction programs. Genes were selected based on their biological relevance to each program and detectability in the spatial transcriptomic dataset.

The ECM/HSC-related fibrogenic score was calculated using the following genes: ACTA2, COL1A1, COL1A2, COL3A1, COL6A3, LAMA2, LAMB1, TAGLN, PDGFRB, and DCN. The hypoxia score was calculated using CA9, VEGFA, ADM, PDK1, LDHA, ENO1, HK2, PGK1, and EGLN3. The progenitor/ductular reaction score was calculated using SOX9, PROM1, KRT19, EPCAM, TACSTD2, and KRT7.

For each spatial spot, module scores were calculated as the average normalized expression of detected genes in each module after z-score standardization. C7 expression and module scores were visualized on the corresponding tissue image to assess their spatial distribution. C7-high and progenitor-high spots were defined based on the upper quantile of the respective expression or module-score distributions and were overlaid on the tissue image to evaluate their spatial relationship.

Supplementary figure legends

Fig. S1. Participant Flow of the UK Biobank Cohort and Additional Validation Analyses of Serum C7.

(A) Flow diagram of participant selection for the UK Biobank cohort used for external validation. Among 43,756 participants with available serum C7 measurements, 4,486 participants with MASLD were included in the final analysis after applying predefined exclusion criteria.

(B) Serum C7 levels in MASLD patients stratified by NAFLD activity score (NAS). Data are shown separately for the main cohort (left, n = 323) and the sub cohort (right, n = 107) (Kruskal–Wallis test; p values are shown in the panel).

(C) Serum C7 levels according to clinically relevant liver stiffness measurement categories (<8, 8–12, 12–20, and ≥20 kPa) in the VCTE cohort (Kruskal–Wallis test; p value is shown in the panel).

(D) Representative double immunofluorescence image of C7 and α-SMA in F4 liver tissue. C7 is shown in red, α-SMA in green, and nuclei are counterstained with DAPI in blue.

Abbreviations: C7, complement component 7; DAPI, 4′,6-diamidino-2-phenylindole; LSM, liver stiffness measurement; MASLD, metabolic dysfunction-associated steatotic liver disease; NAS, NAFLD activity score; VCTE, vibration-controlled transient elastography; α-SMA, alpha-smooth muscle actin.

Fig. S2. C7-Associated Transcriptomic Changes in Endothelial Cells Under MASLD-Cirrhosis Conditions

(A) Top 50 genes positively correlated with C7 expression in endothelial cells under MASLD-cirrhosis, ranked by Pearson correlation coefficient (Pearson correlation analysis).

(B) Top 20 pathways enriched among genes positively correlated with C7 in endothelial cells based on GO and pathway enrichment analysis (pathway enrichment analysis; p values are shown in the panel).

Abbreviations: GO, Gene Ontology.

Fig. S3. Spatial association of C7 expression with fibrotic, progenitor/ductular reaction, and hypoxia-related programs.

(A) Spatial feature maps showing C7 expression, ECM/HSC-related fibrogenic score, hypoxia score, and progenitor/ductular reaction score in MASLD liver tissue.

(B) Spot-level correlation analyses between C7 expression and ECM/HSC-related fibrogenic score, progenitor/ductular reaction score, and hypoxia score. Spearman correlation coefficients and p-values are shown.

(C) Spatial overlap between C7-high spots and progenitor-high spots. C7-high and progenitor-high spots were defined as spots in the upper quartile of C7 expression and progenitor/ductular reaction score, respectively. Red indicates C7-high only spots, blue indicates progenitor-high only spots, and purple indicates overlapping spots.

Abbreviations: C7, complement component 7; ECM, extracellular matrix; HSC, hepatic stellate cell; MASLD, metabolic dysfunction-associated steatotic liver disease.

Fig. S4. Relationship between serum C7 and complement activation intermediates in MASLD.

(A) Correlation between serum C7 and serum C5a levels (Spearman's rank correlation coefficient; p value is shown in the panel).

(B) Correlation between serum C7 and serum soluble C5b-9 levels (Spearman's rank correlation coefficient; p value is shown in the panel).

Fig. S1

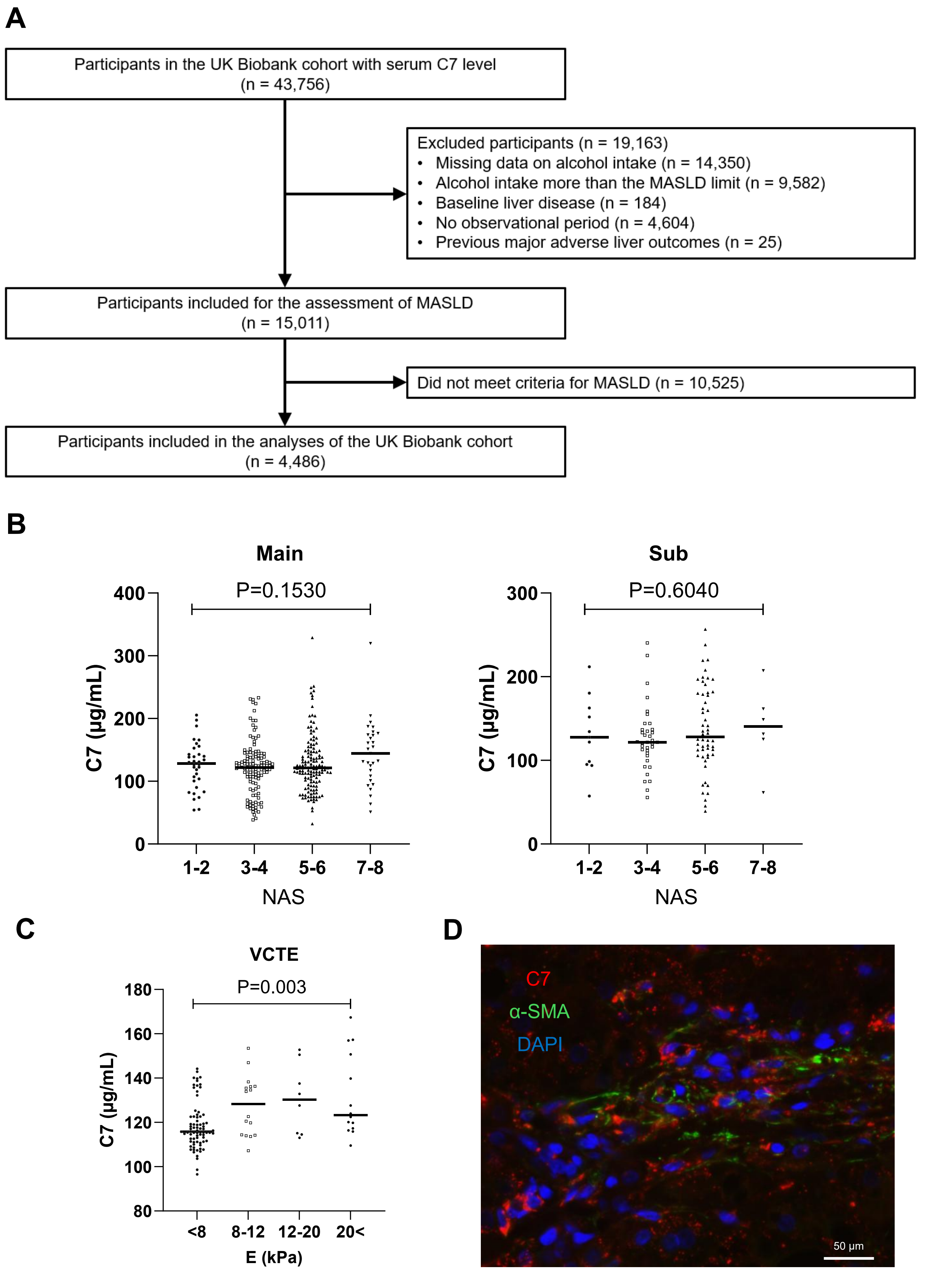

A
Participants in the UK Biobank cohort with serum C7 level
(n = 43,756)
Excluded participants (n = 19,163)
• Missing data on alcohol intake (n = 14,350)
• Alcohol intake more than the MASLD limit (n = 9,582)
• Baseline liver disease (n = 184)
• No observational period (n = 4,604)
• Previous major adverse liver outcomes (n = 25)
Participants included for the assessment of MASLD
(n = 15,011)
Did not meet criteria for MASLD (n = 10,525)
Participants included in the analyses of the UK Biobank cohort
(n = 4,486)
B
Main
P=0.1530
C7 (μg/mL)
400
300
200
100
0
1-2
3-4
5-6
7-8
NAS
Sub
P=0.6040
C7 (μg/mL)
300
200
100
0
1-2
3-4
5-6
7-8
NAS
C
VCTE
P=0.003
C7 (μg/mL)
180
160
140
120
100
80
<8
8-12
12-20
20<
E (kPa)
D
C7
α-SMA
DAPI
50 μm

Fig. S2

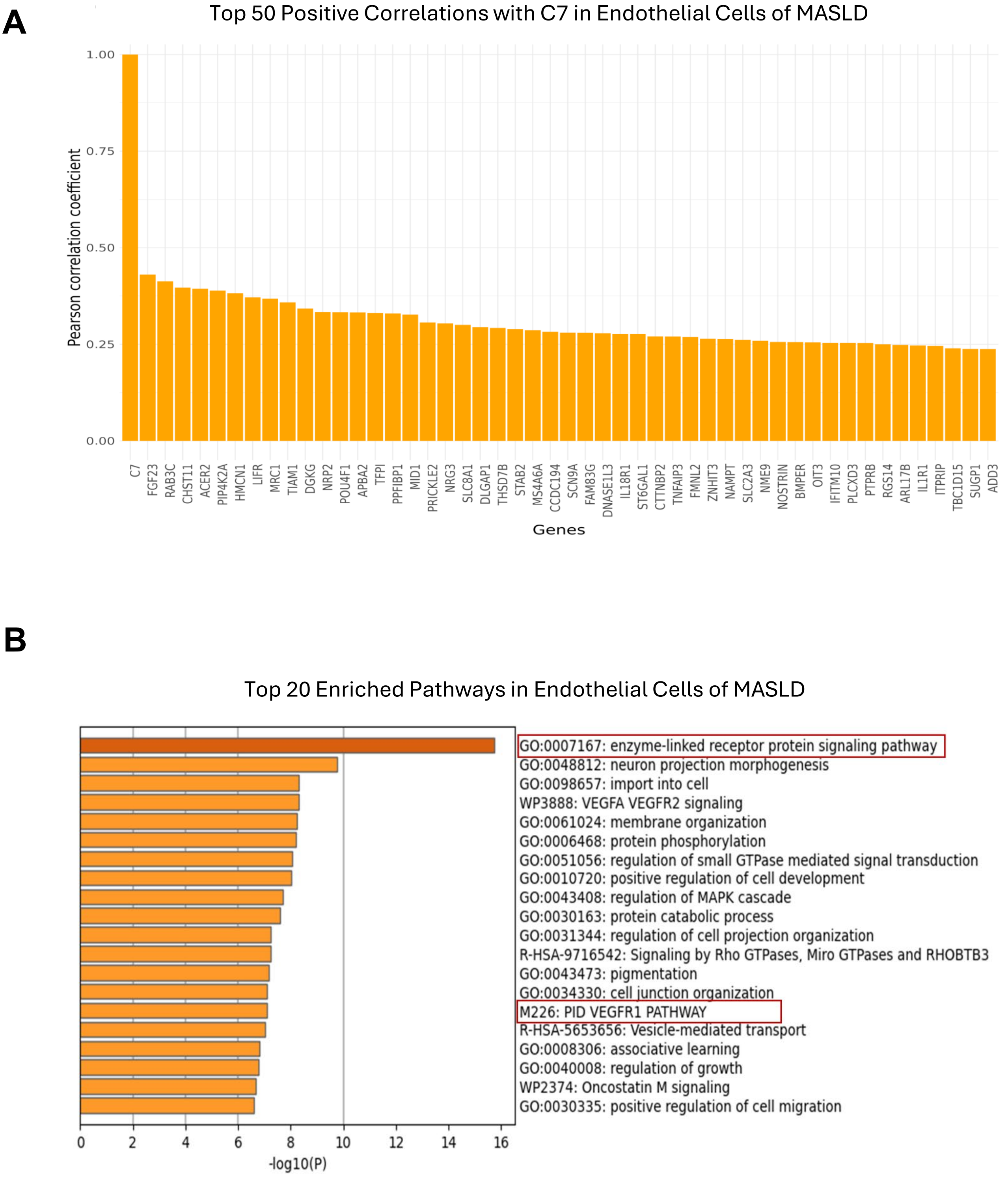
A
Top 50 Positive Correlations with C7 in Endothelial Cells of MASLD
Pearson correlation coefficient
1.00
0.75
0.50
0.25
0.00
C7
FGF23
RAB3C
CHST11
ACER2
PIP4K2A
HMCN1
LIFR
MRC1
TIAM1
DGKG
NRP2
POU4F1
APBA2
TFPI
PPFIBP1
MID1
PRICKLE2
NRG3
SLC8A1
DLGAP1
THSD7B
STAB2
MS4A6A
CCDC194
SCN9A
FAM83G
DNASE1L3
IL18R1
ST6GAL1
CTTNBP2
TNFAIP3
FMNL2
ZNHIT3
NAMPT
SLC2A3
NME9
NOSTRIN
BMPER
OIT3
IFITM10
PLCXD3
PTPRB
RGS14
ARL17B
IL1R1
ITPRIP
TBC1D15
SUGP1
ADD3
Genes
B
Top 20 Enriched Pathways in Endothelial Cells of MASLD
GO:0007167: enzyme-linked receptor protein signaling pathway
GO:0048812: neuron projection morphogenesis
GO:0098657: import into cell
WP3888: VEGFA VEGFR2 signaling
GO:0061024: membrane organization
GO:0006468: protein phosphorylation
GO:0051056: regulation of small GTPase mediated signal transduction
GO:0010720: positive regulation of cell development
GO:0043408: regulation of MAPK cascade
GO:0030163: protein catabolic process
GO:0031344: regulation of cell projection organization
R-HSA-9716542: Signaling by Rho GTPases, Miro GTPases and RHOBTB3
GO:0043473: pigmentation
GO:0034330: cell junction organization
M226: PID VEGFR1 PATHWAY
R-HSA-5653656: Vesicle-mediated transport
GO:0008306: associative learning
GO:0040008: regulation of growth
WP2374: Oncostatin M signaling
GO:0030335: positive regulation of cell migration
0
2
4
6
8
10
12
14
16
-log10(P)

Fig. S3

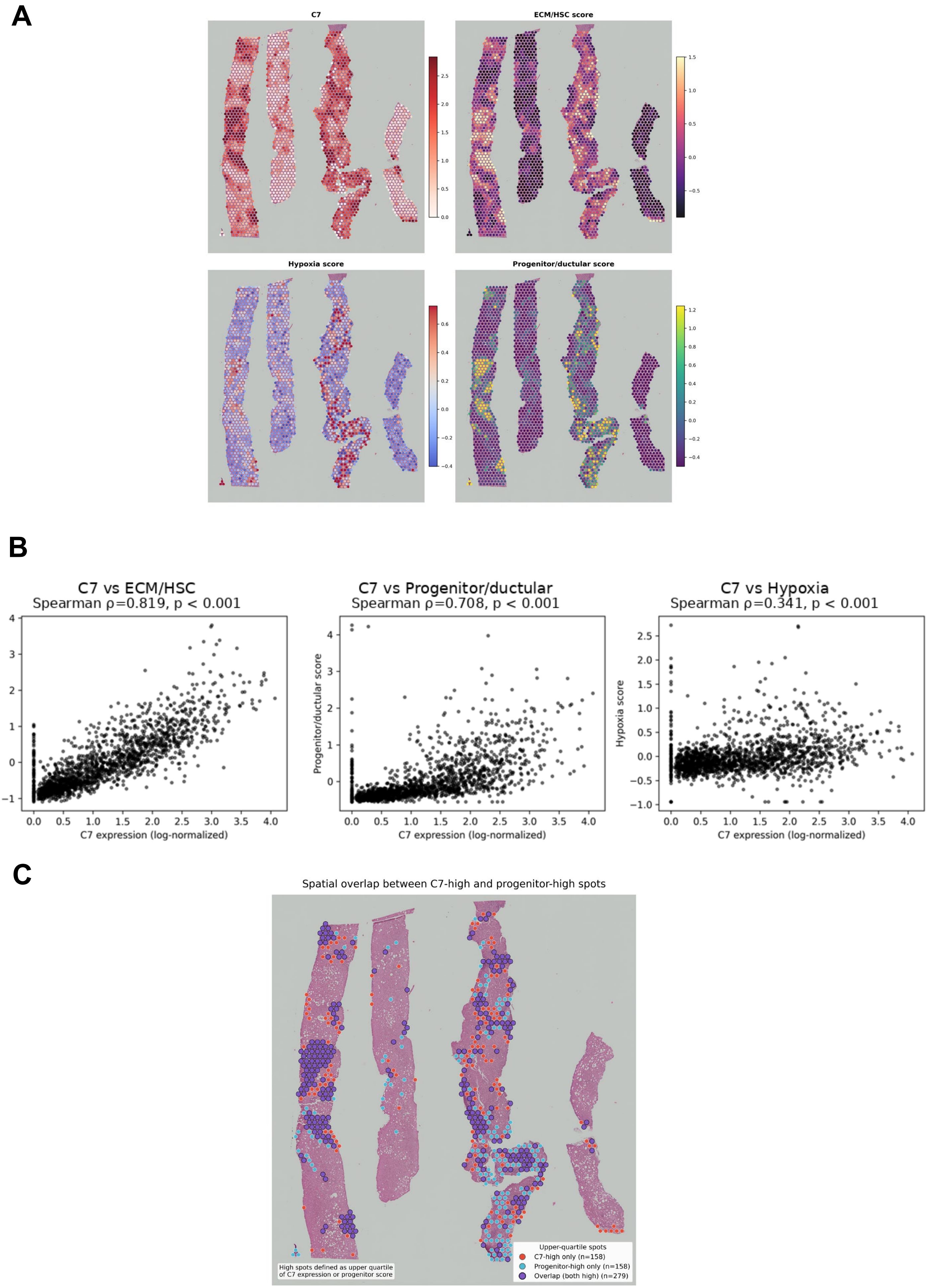
A
C7
ECM/HSC score
Hypoxia score
Progenitor/ductular score
B
C7 vs ECM/HSC
Spearman ρ=0.819, p < 0.001
ECM/HSC score
C7 expression (log-normalized)
C7 vs Progenitor/ductular
Spearman ρ=0.708, p < 0.001
Progenitor/ductular score
C7 expression (log-normalized)
C7 vs Hypoxia
Spearman ρ=0.341, p < 0.001
Hypoxia score
C7 expression (log-normalized)
C
Spatial overlap between C7-high and progenitor-high spots
High spots defined as upper quartile of C7 expression or progenitor score
Upper-quartile spots
C7-high only (n=158)
Progenitor-high only (n=158)
Overlap (both high) (n=279)

Fig. S4

A

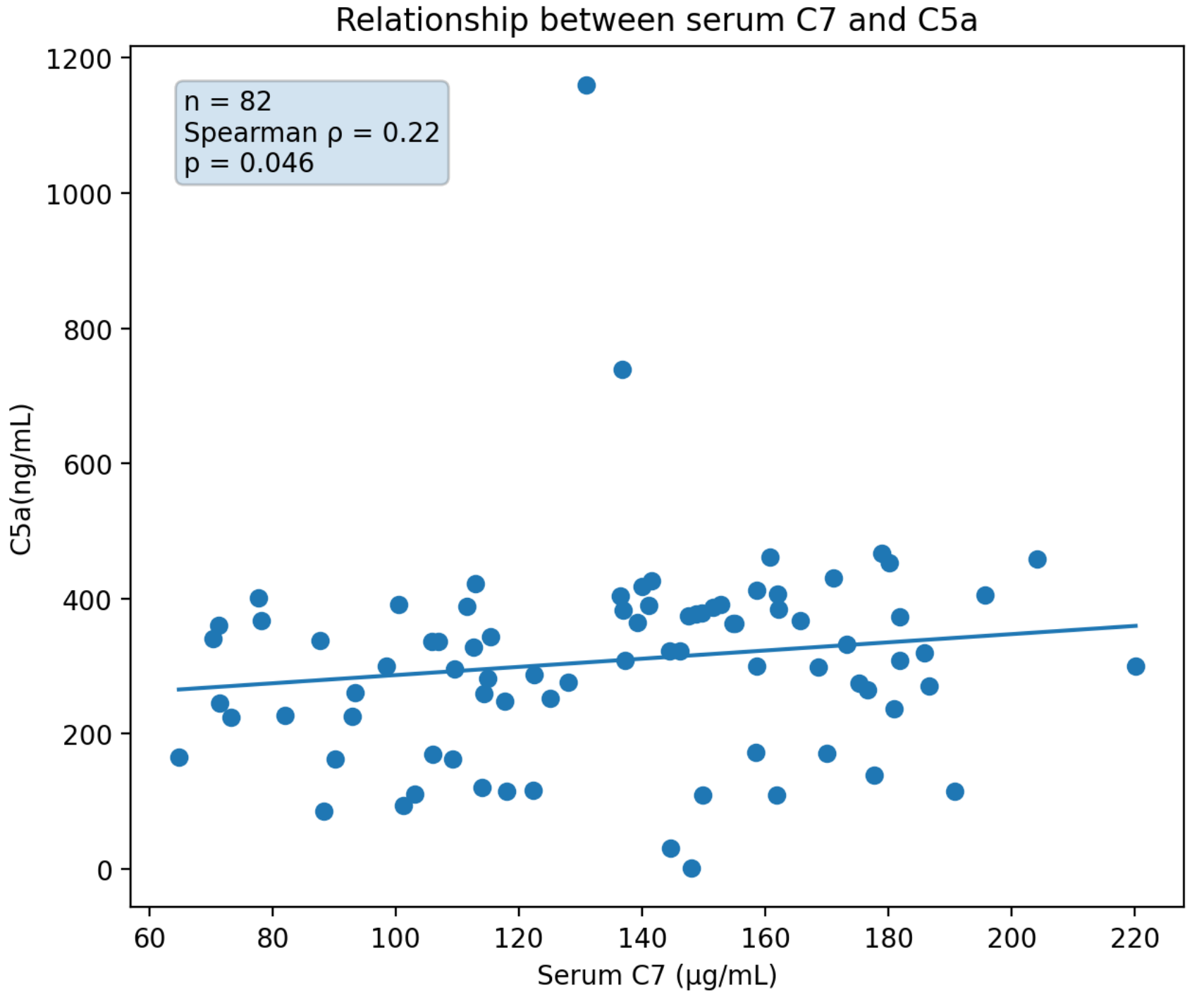
Relationship between serum C7 and C5a
n = 82
Spearman ρ = 0.22
p = 0.046
C5a(ng/mL)
0
200
400
600
800
1000
1200
60
80
100
120
140
160
180
200
220
Serum C7 (μg/mL)

B

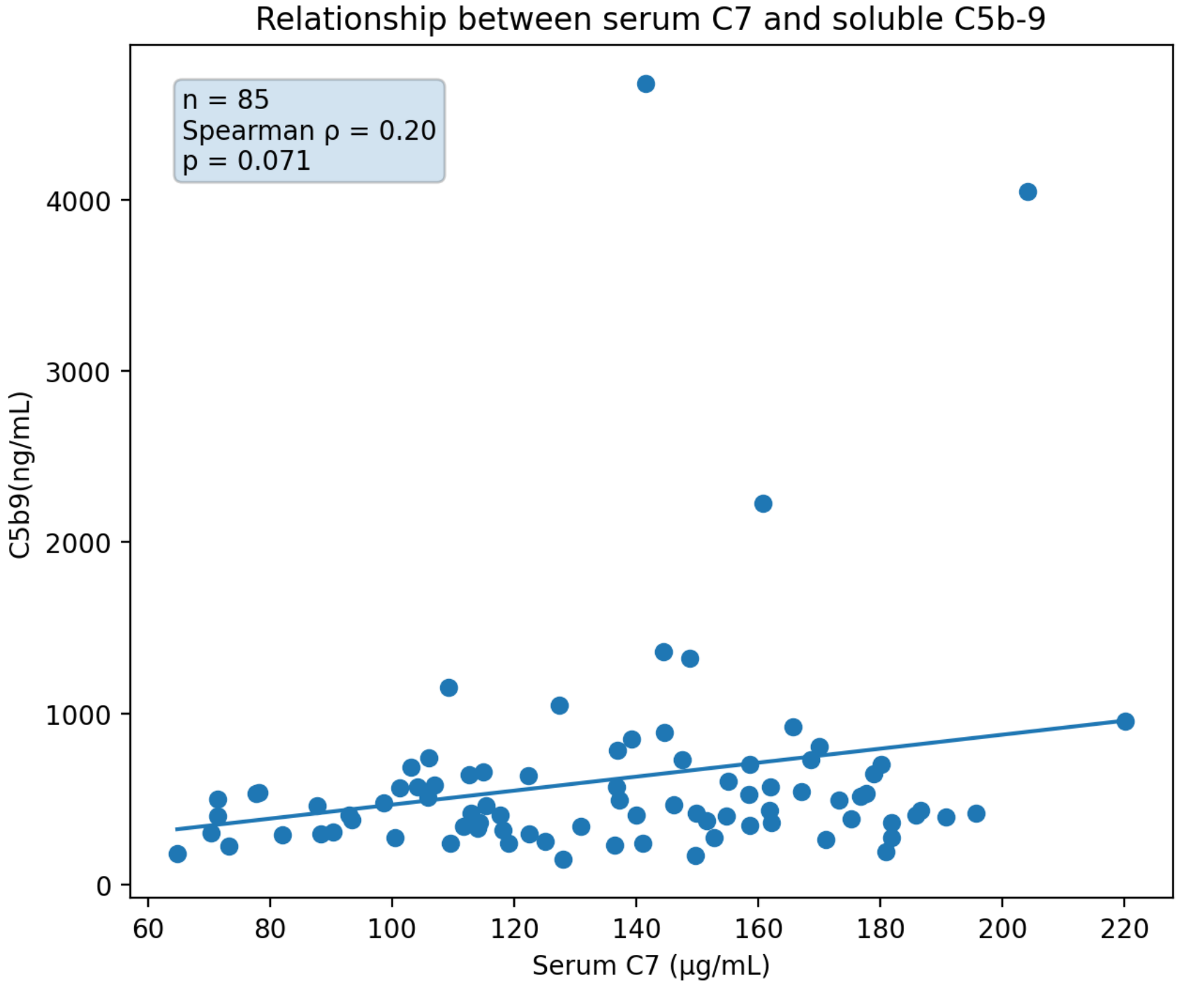
Relationship between serum C7 and soluble C5b-9
n = 85
Spearman ρ = 0.20
p = 0.071
C5b9(ng/mL)
0
1000
2000
3000
4000
60
80
100
120
140
160
180
200
220
Serum C7 (μg/mL)

Table S1. Baseline Characteristics of MASLD Patients in the Biopsy Cohort

	Median (IQR)/n (%)		
	**Main cohort (n = 323)**	**Sub cohort (n = 107)**	**p–value**
Age (years)	56 (15–84)	57 (16–77)	0.434
Male	142 (44)	45 (42)	0.816
BMI (kg/m^2)	26.6 (17.6–45.1)	27.1 (18.3–39.7)	0.511
DM	119 (37)	42 (39)	0.740
HT	126 (39)	51 (48)	0.143
DL	205 (63)	69 (64)	0.941
**Laboratory data**			
Albumin (g/dL)	4.5 (1.8–5.4)	4.5 (3.4–5.2)	0.805
T-Bil (U/L)	0.88 (0.3–4.63)	0.89 (0.4–1.91)	0.892
AST (U/L)	48 (13–263)	49 (17–230)	0.871
ALT (U/L)	71 (13–522)	71 (15–618)	0.836
γ-GT (U/L)	57 (7–1120)	54 (21–578)	0.292
BUN (mg/dL)	13.0 (5.0–27.5)	13.2 (5.0–23.4)	0.389
Cre (mg/dL)	0.67 (0.32–1.39)	0.69 (0.47–1.37)	0.158
TC (mg/dL)	207 (70–336)	206 (113–289)	0.266
TG (mg/dL)	126 (39–801)	122 (32–368)	0.351
LDL-C (mg/dL)	128 (68–246)	126 (44–205)	0.713
HDL-C (mg/dL)	51 (13–114)	51 (23–81)	0.882
Plt (×10^4/μL)	22.4 (5.3–86.1)	21.5 (6.3–39)	0.686
HbA1c (%)	5.7 (3.5–11.9)	5.7 (4.4–13.5)	0.970
FBG (mg/dL)	107 (74–391)	105 (7–196)	0.268
HOMA-IR	3.44 (0.15–45.95)	3.44 (0.23–37.18)	0.998
AFP (ng/mL)	3.4 (0.7–307)	3.25 (1.2–13.2)	0.897
C7 (μg/mL)	124.1 (32.2–328.8)	125.2 (39.1–256.7)	0.247
**Histological findings**			
Fibrosis (F0/1/2/3/4)	55/138/39/71/20	18/46/13/24/6	0.999
Advanced fibrosis (≥F3)	91 (28)	31 (28)	0.999
Steatosis (1/2/3)	109/130/84	31/59/17	**0.043**
Lobular inflammation (0/1/2/3)	12/173/119/19	7/42/51/7	0.066
Ballooning (0/1/2)	66/163/94	22/61/24	0.374
NAS score ≥5	166 (51)	60 (57)	0.466

Statistical analysis: Continuous variables were compared using the Mann–Whitney U test, and categorical variables were compared using the chi-square test or Fisher’s exact test, as appropriate. Exact p values are shown.

Abbreviations: AFP, alpha-fetoprotein; ALT, alanine aminotransferase; AST, aspartate aminotransferase; BMI, body mass index; BUN, blood urea nitrogen; C7, complement component 7; Cre, creatinine; DL, dyslipidemia; DM, diabetes mellitus; F, fibrosis stage; FBG, fasting blood glucose; γ-GT, gamma-glutamyltransferase; HbA1c, hemoglobin A1c; HDL-C, high-density lipoprotein cholesterol; HOMA-IR, homeostasis model assessment of insulin resistance; HT, hypertension; IQR, interquartile range; LDL-C, low-density lipoprotein cholesterol; MASLD, metabolic dysfunction-associated steatotic liver disease; NAS, non-alcoholic fatty liver disease activity score; Plt, platelet count; T-Bil, total bilirubin; TC, total cholesterol; TG, triglycerides.

Table S2. Baseline Characteristics of MASLD Patients in the Follow-Up Cohort

	Median (IQR)/n (%) Follow-up cohort (n = 348)
Age (years)	56 (15–84)
Male	141 (41)
BMI (kg/m^2)	25.5 (17.6–45.1)
DM	142 (41)
HT	151 (43)
DL	214 (62)
**Laboratory data**	
Albumin (g/dL)	4.5 (3.0–5.4)
T-Bil (U/L)	0.88 (0.4–3.31)
AST (U/L)	49 (13–263)
ALT (U/L)	71 (14–618)
γ-GT (U/L)	58 (7–1120)
BUN (mg/dL)	13.0 (5.0–27.5)
Cre (mg/dL)	0.66 (0.32–1.39)
TC (mg/dL)	205 (70–336)
TG (mg/dL)	123 (32–801)
LDL-C (mg/dL)	123 (44–246)
HDL-C (mg/dL)	51 (13–97)
Plt (×10^4/μL)	21.6 (6.3–86.1)
HbA1c (%)	5.7 (3.5–11.9)
FBG (mg/dL)	108 (74–391)
HOMA-IR	3.3 (0.15–45.95)
AFP (ng/mL)	3.2 (0.7–307)
C7 (μg/mL)	123.9 (32.2–328.8)
**Histological findings**	
Fibrosis (F0/1/2/3/4)	18/46/13/24/6
Advanced fibrosis (≥F3)	102 (29)
Steatosis (1/2/3)	115/152/81
Lobular inflammation (0/1/2/3)	15/171/142/20
Ballooning (0/1/2)	66/183/99
NAS score ≥5	185 (53)

Abbreviations: AFP, alpha-fetoprotein; ALT, alanine aminotransferase; AST, aspartate aminotransferase; BMI, body mass index; BUN, blood urea nitrogen; C7, complement component 7; Cre, creatinine; DL, dyslipidemia; DM, diabetes mellitus; F, fibrosis stage;

FBG, fasting blood glucose; γ-GT, gamma-glutamyltransferase; HbA1c, hemoglobin A1c; HDL-C, high-density lipoprotein cholesterol; HOMA-IR, homeostasis model assessment of insulin resistance; HT, hypertension; IQR, interquartile range; LDL-C, low-density lipoprotein cholesterol; MASLD, metabolic dysfunction-associated steatotic liver disease; NAS, non-alcoholic fatty liver disease activity score; Plt, platelet count; T-Bil, total bilirubin; TC, total cholesterol; TG, triglycerides.

Table S3. Baseline Characteristics of MASLD Patients in the VCTE Cohort

	Median (IQR)/n (%)<br>VCTE cohort<br>(n = 120)
Age (years)	59 (16–86)
Male	66 (55)
BMI (kg/m^2)	26.2 (19.2–39.4)
DM	43 (35.8)
HT	48 (40.0)
DL	47 (39.2)
**Laboratory data**	
Albumin (g/dL)	4.4 (3.0–5.3)
T-Bil (U/L)	0.80 (0.30–5.0)
AST (U/L)	32 (15–155)
ALT (U/L)	40 (11–338)
γ-GT (U/L)	44 (12–582)
BUN (mg/dL)	15.0 (5.8–46.7)
Cre (mg/dL)	0.8 (0.5–4.24)
TC (mg/dL)	197 (86–307)
TG (mg/dL)	128 (45–664)
LDL-C (mg/dL)	113 (37–177)
HDL-C (mg/dL)	49 (21–96)
Plt (×10^4/μL)	20.8 (4.6–41.6)
HbA1c (%)	6.0 (4.3–8.3)
FBG (mg/dL)	110 (72–342)
AFP (ng/mL)	3.3 (1.2–11.3)
C7 (μg/mL)	118.1 (96.6–255.7)
**Fibroscan findings**	
LSM (kPa)	6.1 (3.0–66.7)

Abbreviations: AFP, alpha-fetoprotein; ALT, alanine aminotransferase; AST, aspartate aminotransferase; BMI, body mass index; BUN, blood urea nitrogen; C7, complement component 7; Cre, creatinine; DL, dyslipidemia; DM, diabetes mellitus; FBG, fasting blood glucose; γ-GT, gamma-glutamyltransferase; HbA1c, hemoglobin A1c; HDL-C, high-density lipoprotein cholesterol; HT, hypertension; IQR, interquartile range; LDL-C, low-density lipoprotein cholesterol; LSM, liver stiffness measurement; MASLD, metabolic dysfunction-associated steatotic liver disease; Plt, platelet count; T-Bil, total bilirubin; TC, total cholesterol; TG, triglycerides.

Table S4. Multiple regression analysis for factors associated with liver fibrosis in the Main cohort

	Univariate			Multivariable		
	**stdβ**	**95% CI**	**p-value**	**stdβ**	**95% CI**	**p-value**
Age	0.37	0.28–0.47	**< 0.001**	0.45	0.34–0.55	**< 0.001**
Male	-0.16	-0.27–-0.060	**0.002**	-0.023	-0.11–0.087	0.85
BMI	0.22	0.11–0.32	**< 0.001**	0.28	0.19–0.38	**< 0.001**
DM	0.19	0.087–0.30	**< 0.001**	0.061	-0.033–0.15	0.20
ALT	0.12	0.016–0.23	**0.024**	0.18	0.088–0.28	**< 0.001**
C7	0.23	0.13–0.33	**< 0.001**	0.13	0.035–0.22	**0.007**

Statistical analysis: Univariable and multivariable linear regression analyses were performed. Standardized beta coefficients, 95% confidence intervals, and exact p values are shown.

Abbreviations: ALT, alanine aminotransferase; BMI, body mass index; C7, complement component 7; CI, confidence interval; DM, diabetes mellitus; stdβ, standardized beta coefficient.

Table S5. Correlation Between Serum C7 Levels and Clinicopathological Parameters in the Main Cohort

	Correlation coefficient	p-value
Plt	-0.305	**< 0.001**
AFP	0.269	**< 0.001**
Fibrosis	0.232	**< 0.001**
Age	0.203	**< 0.001**
AST	0.153	0.006
Albumin	-0.126	0.024
γ-GT	0.113	0.042
Lobular inflammation	0.107	0.055
LDL-C	-0.092	0.122
HbA1c	0.088	0.119
HOMA-IR	0.087	0.131
Ballooning	0.077	0.165
TG	0.065	0.242
NAS	0.052	0.340
Steatosis	-0.047	0.398
BUN	0.041	0.472
FBG	0.039	0.483
ALT	0.037	0.507
BMI	-0.032	0.566
T-Bil	0.017	0.763
HDL-C	0.016	0.775
TC	0.001	0.982

Statistical analysis: Correlations were assessed using Spearman's rank correlation coefficient. Correlation coefficients and exact p values are shown. Table entries are listed in order of strongest correlation.

Abbreviations: AFP, alpha-fetoprotein; ALT, alanine aminotransferase; AST, aspartate aminotransferase; BMI, body mass index; BUN, blood urea nitrogen; C7, complement component 7; FBG, fasting blood glucose; γ-GT, gamma-glutamyltransferase; HbA1c, hemoglobin A1c; HDL-C, high-density lipoprotein cholesterol; HOMA-IR, homeostasis model assessment of insulin resistance; LDL-C, low-density lipoprotein cholesterol; NAS, non-alcoholic fatty liver disease activity score; Plt, platelet count; T-Bil, total bilirubin; TC, total cholesterol; TG, triglycerides.

Table S6. Clinical Characteristics of MASLD Patients with and Without LREs in the Follow-Up Cohort

	Median (IQR)/n (%)		
	**No LRE (n = 326)**	**LRE (n = 22)**	**p-value**
Age (years)	55 (15–88)	65 (37–84)	**< 0.001**
Male	135 (41)	6 (27)	0.191
BMI (kg/m^2)	26.5 (17.6–45.1)	27.3 (18.3–30.3)	0.865
DM	127 (39)	15 (68)	**0.007**
HT	134 (41)	17 (77)	**< 0.001**
DL	206 (63)	8 (36)	**0.012**
**Laboratory data**			
Albumin (g/dL)	4.5 (3.0–5.4)	4.1 (3.5–4.7)	**< 0.001**
T-Bil (U/L)	0.88 (0.3–3.31)	1.03 (0.44–2.15)	0.578
AST (U/L)	48 (13–263)	53 (31–120)	0.365
ALT (U/L)	72 (14–618)	53 (16–184)	**0.031**
γ-GT (U/L)	55 (7–1120)	87 (28–257)	**0.004**
BUN (mg/dL)	13.0 (5.0–27.5)	13.5 (7.5–21.3)	0.555
Cre (mg/dL)	0.67 (0.32–1.37)	0.61 (0.4–1.1)	0.132
TC (mg/dL)	208 (70–336)	176 (131–247)	**< 0.001**
TG (mg/dL)	124 (32–801)	118 (66–228)	0.503
LDL-C (mg/dL)	128 (44–246)	99 (68–156)	**< 0.001**
HDL-C (mg/dL)	52 (13–97)	49 (35–69)	0.170
Plt (×10^4/μL)	22.1 (7.0–86.1)	11.0 (6.3–18.6)	**< 0.001**
HbA1c (%)	5.7 (3.5–11.9)	6.0 (4.8–10.9)	0.086
FBG (mg/dL)	107 (74–391)	125 (77–175)	**0.012**
HOMA-IR	3.34 (0.15–45.95)	6.87 (1.17–14.41)	**< 0.001**
AFP (ng/mL)	3.3 (0.7–307)	6.1 (1.9–20.3)	**< 0.001**
C7 (μg/mL)	122.1 (32.2–256.7)	162.1 (118.6–328.8)	**< 0.001**
**Histological findings**			
Fibrosis (F0/1/2/3/4)	57/147/38/73/11	0/1/3/6/12	**< 0.001**
Advanced fibrosis (≥F3)	84 (25.8)	18 (82)	**< 0.001**
Steatosis (1/2/3)	101/145/80	15/6/1	**0.004**
Lobular inflammation (0/1/2/3)	15/166/128/17	0/5/14/3	**0.020**
Ballooning (0/1/2)	65/171/90	23/50/35	0.144
NAS score ≥5	168 (52)	12 (55)	0.455

Statistical analysis: Continuous variables were compared using the Mann–Whitney U test, and categorical variables were compared using the chi-square test or Fisher’s exact test, as appropriate. Exact p values are shown.

Abbreviations: AFP, alpha-fetoprotein; ALT, alanine aminotransferase; AST, aspartate aminotransferase; BMI, body mass index; BUN, blood urea nitrogen; C7, complement component 7; Cre, creatinine; DL, dyslipidemia; DM, diabetes mellitus; F, fibrosis stage; FBG, fasting blood glucose; γ-GT, gamma-glutamyltransferase; HbA1c, hemoglobin A1c; HDL-C, high-density lipoprotein cholesterol; HOMA-IR, homeostasis model assessment of insulin resistance; HT, hypertension; IQR, interquartile range; LDL-C, low-density lipoprotein cholesterol; MASLD, metabolic dysfunction-associated steatotic liver disease; NAS, non-alcoholic fatty liver disease activity score; Plt, platelet count; T-Bil, total bilirubin; TC, total cholesterol; TG, triglycerides.

Table S7. Cox Proportional Hazards Models for LRE Risk in the Follow-Up Cohort

	Univariate			Multivariable		
	**HR**	**95%CI**	**p-value**	**HR**	**95%CI**	**p-value**
Age ≥61 years	3.73	1.52–9.15	**0.004**	1.78	0.71–4.46	0.222
DM	2.95	1.20–7.24	**0.018**	2.20	0.89–5.44	0.088
Fibrosis ≥F3	13.78	4.63–41.01	**< 0.001**	7.83	2.53–24.21	**< 0.001**
C7 ≥140	7.72	2.60–22.95	**< 0.001**	4.54	1.49–13.82	**0.008**

Statistical analysis: Univariable and multivariable Cox proportional hazards regression analyses were performed. Hazard ratios, 95% confidence intervals, and exact p values are shown.

Abbreviations: C7, complement component 7; CI, confidence interval; DM, diabetes mellitus; HR, hazard ratio; LRE, liver-related event.

Table S8. Baseline Characteristics of Participants with MASLD in the UK Biobank Cohort According to C7 Status

Characteristics	Overall (N = 4,486)	Low C7 (n = 2,243)	High C7 (n = 2,243)
Age, years	60 [53–64]	58 [51–63]	61 [55–65]
Female sex	3067 (68.4)	1549 (69.1)	1518 (67.7)
Male sex	1419 (31.6)	694 (30.9)	725 (32.3)
BMI, kg/m^2	30.2 [28.1–33.0]	30.0 [28.0–32.8]	30.4 [28.3–33.1]
Type 2 diabetes	484 (10.8)	140 (6.2)	344 (15.3)
Ethnicity			
White	4350 (97.2)	2185 (97.6)	2165 (96.8)
Asian	39 (0.9)	25 (1.1)	14 (0.6)
Others	86 (1.9)	29 (1.3)	57 (2.5)
Smoking status			
Never	2309 (51.5)	1225 (54.6)	1084 (48.3)
Former	1830 (40.8)	839 (37.4)	991 (44.2)
Current	347 (7.7)	179 (8.0)	168 (7.5)
Alcohol intake, g/day	13.3 [8.3–19.5]	13.5 [8.4–19.7]	13.0 [7.7–19.2]
CMRFs			
Overweight or obesity	4401 (98.1)	2203 (98.2)	2198 (98.0)
Dysglycemia or type 2 diabetes mellitus	1542 (34.4)	607 (27.1)	935 (41.7)
Hypertriglyceridemia	3146 (70.1)	1568 (69.9)	1578 (70.4)
Low HDL-cholesterol	1817 (40.5)	837 (37.3)	980 (43.7)
Hypertension	3604 (80.3)	1742 (77.7)	1862 (83.0)
ALT, IU/L	25.7 [19.7–34.2]	24.7 [19.2–32.9]	26.8 [20.4–35.7]
AST, IU/L	26.1 [22.6–31.1]	25.3 [21.9–29.7]	27.1 [23.5–32.5]
GGT, IU/L	37.3 [27.2–55.7]	35.8 [26.4–52.7]	39.1 [28.2–58.6]
Total cholesterol, mmol/L	5.7 [4.83–6.53]	5.8 [4.9–6.57]	5.6 [4.7–6.5]
HDL-cholesterol, mmol/L	1.2 [1.04–1.40]	1.2 [1.0–1.4]	1.2 [1.0–1.4]
LDL-cholesterol, mmol/L	3.6 [2.97–4.28]	3.7 [3.1–4.3]	3.6 [2.9–4.2]
Triglycerides, mmol/L	2.1 [1.59–2.88]	2.1 [1.6–2.9]	2.1 [1.58–2.88]
Albumin, g/L	45.2 [43.48–46.95]	45.2 [43.4–46.9]	45.2 [43.5–47.0]
Platelet count, 10^9/L	244 [210–282]	246 [213–282]	242 [207–281]
HbA1c, mmol/L	36.2 [33.6–39.2]	35.5 [33.1–38.0]	37.0 [34.2–40.2]
Glucose, mmol/L	4.99 [4.65–5.41]	4.95 [4.62–5.31]	5.04 [4.68–5.56]
FIB-4	1.24 [0.98–1.58]	1.18 [0.94–1.48]	1.31 [1.03–1.69]

<1.3	2492 (55.6)	1390 (62.0)	1102 (49.1)
1.3-2.67	1879 (41.9)	826 (36.8)	1053 (46.9)
>2.67	115 (2.6)	27 (1.2)	88 (3.9)
C7, NPX	-0.04 [-0.22–0.15]	-0.22 [-0.38–-0.12]	0.15 [0.04–0.29]

Values are presented as medians (interquartile ranges) or numbers (percentages).

Participants were classified into low and high C7 groups based on the median serum C7 level of the cohort.

Serum C7 was measured using the Olink® proximity extension assay and is expressed as NPX ($\log_2$ scale).

Abbreviations: ALT, alanine aminotransferase; AST, aspartate aminotransferase; BMI, body mass index; C7, complement component 7; CMRF, cardiometabolic risk factor; FIB-4, fibrosis-4 index; GGT, gamma-glutamyl transferase; HbA1c, hemoglobin A1c; HDL-cholesterol, high-density lipoprotein cholesterol; LDL-cholesterol, low-density lipoprotein cholesterol; NPX, normalized protein expression.

Table S9. ICD-10 Codes Used to Identify Previous Chronic Liver Diseases

Liver diseases	ICD codes
α1-antitrypsin deficiency	E88.0
Autoimmune hepatitis	K75.4
Budd–Chiari syndrome	I82.0, K76.5
Hemochromatosis	E83.1
Primary biliary cholangitis	K74.3, K74.5
Primary sclerosing cholangitis	K83.0 (+ K50 or K51)
Wilson disease	E83.0
Viral hepatitis	B15–B19

ICD10, International Classification of Diseases, 10th Revision

Table S10. ICD-10 Codes Used to Identify Major Adverse Liver Outcomes

Diagnosis	ICD-10 codes
Cirrhosis	K70.2, K70.3, K74.0, K74.6, K76.6
Varices	I85.0, I85.1, I85.9, I86.4, I98.2, I98.3
Hepatocellular carcinoma	C22.0
Liver failure	K70.4, K72.0, K72.1, K72.9, K76.7
Liver transplantation	Z94.4, K72.9
Other liver diseases	C22.7, C22.8, K70.9, K73.0, K73.1, K73.2, K73.8, K73.9, K75.8, K75.9

Abbreviations: ICD10, International Classification of Diseases, 10th Revision

Table S11. Pure Alcohol Content from Beverage-Specific Serving Sizes

Beverage	Typical volume (mL)	alcohol-by-volume (%)	Alcohol per unit (g)
Red wine	125 (per glass)	12	11.8
White wine	125 (per glass)	12	11.8
Beer / cider	568 (per pint)	5	22.4
Spirits	25 (per measure)	40	7.9
Fortified wine	50 (per glass)	17	6.7

Pure alcohol content was derived from beverage-specific serving sizes, alcohol-by-volume values, and the density of ethanol using the following formula: alcohol (g) = volume (mL) × alcohol-by-volume values (expressed as a decimal fraction) × 0.789.

Table S12. Distribution of Missing Data and Multiple Imputation

Variables	Count	Percentage
Glucose	2009	14.2
HDL-cholesterol	1993	13.3
Albumin	1983	13.2
SBP	904	6.02
DBP	904	6.02
AST	781	5.20
LDL	759	5.06
HbA1c	754	5.02
Triglycerides	747	4.98
GGT	747	4.98
ALT	741	4.94
Total cholesterol	741	4.94
Platelet	475	3.16
BMI	46	0.31
Weight	38	0.25
Height	34	0.23

Among participants assessed for MASLD (n = 15,011), missing values in covariates other than C7 were handled using multiple imputation by chained equations implemented with the mice package in R (five imputations, m = 5; five iterations, maxit = 5).

Abbreviations: ALT, alanine aminotransferase; AST, aspartate aminotransferase; BMI, body mass index; DBP, diastolic blood pressure; GGT, gamma-glutamyl transferase; HbA1c, Hemoglobin A1c; HDL-cholesterol; high-density lipoprotein cholesterol; LDL, low-density lipoprotein; SBP, systolic blood pressure

Table S13. Multivariable Model for Predicting Major Adverse Liver Outcomes in the UK Biobank Cohort

Variables	HR (95% CI)	*p*-value
Age	1.03 (1.00–1.06)	0.058
Female	0.90 (0.61–1.33)	0.609
DM	1.08 (0.65–1.81)	0.756
FIB-4	1.18 (1.04–1.34)	**0.012**
C7 (per 1 NPX)	7.47 (4.63–12.07)	**<0.001**

Statistical analysis: Multivariable Cox proportional hazards regression analysis was performed. Hazard ratios, 95% confidence intervals, and exact p values are shown.

Abbreviations: C7, complement component 7; CI, confidence interval; DM, diabetes mellitus; FIB-4, fibrosis-4 index; HR, hazard ratio; NPX, normalized protein expression.
