# Supplementary material for: Integrative multi-omics analysis identifies stellate cell-derived complement component 7 as a predictor of fibrosis and liver-related events in MASLD
Source: JHEP Rep. 2026 Jul 22;8(11):101965. doi: 10.1016/j.jhepr.2026.101965 (PMC13651563; doi:10.1016/j.jhepr.2026.101965)
Supplement: Supplementary file 2 [file mmc2.pdf]

Supplementary CTAT Methods Table

Integrative Multi-Omics Analysis Identifies Stellate Cell-Derived Complement Component 7 as a Predictor of Fibrosis and Liver-Related Events in MASLD Patients

1. Study design, cohorts, ethics, and specimens

Category	Resource / item	Source / identifier	Use / key details
Study design	Retrospective observational multi-cohort biomarker study	Shinshu University School of Medicine; ethics approval no. 5797	Biopsy-confirmed MASLD cohort, follow-up cohort, VCTE cohort, multi-omics discovery, and external validation.
Human participants	Biopsy-confirmed MASLD cohort	Shinshu University Hospital; n=459 initially enrolled; serum-available n=430; Jan 1998-May 2022	Liver biopsy cohort used for serum C7 evaluation and histological assessment.
Human participants	Main and sub cohorts	Main cohort n=323; sub cohort n=107	Biomarker evaluation and performance assessment.
Human participants	Follow-up cohort	n=348; at least one year of follow-up	Longitudinal evaluation of liver-related events (LREs).
Human participants	VCTE cohort	n=120; recruited Sep 2014-Mar 2024	Validation using FibroScan-based liver stiffness measurement.
Human participants	UK Biobank MASLD cohort	REC 11/NW/0382; application no. 930515; final MASLD analysis n=4,486	External validation using Olink-measured plasma C7 and registry-based major adverse liver outcomes (MALO).
Consent	Participant consent	Shinshu University and UK Biobank	Informed consent obtained from participants.
Corresponding methods author	Takefumi Kimura, MD, PhD	Department of Medicine, Division of Gastroenterology and Hepatology, Shinshu University School of Medicine, Asahi 3-1-1, Matsumoto, Nagano 390-8621, Japan. Tel: 81-263-37-2634. Email: kimuratakefumii@yahoo.co.jp; t_kimura@shinshu-u.ac.jp	Author responsible for correspondence regarding methods, materials, resources, and analytical details described in the manuscript.

2. Reagents, antibodies, probes, and assays

Category	Resource / item	Source / identifier	Use / key details
Commercial assay	C7 Human ELISA Kit	Hycult Biotech	Measurement of serum C7 concentrations.
Commercial assay	Human Complement C5a ELISA Kit	Invitrogen	Measurement of serum C5a concentrations.
Commercial assay	Human Complement C5b-9 [soluble] ELISA Kit	Invitrogen	Measurement of soluble C5b-9 concentrations.
RNA extraction	RNeasy Mini Kit	Qiagen, Hilden, Germany	Total RNA extraction from liver biopsy samples and cultured cells.
Reverse transcription	PrimeScript RT Reagent Kit	Takara Bio, Shiga, Japan	cDNA synthesis for qPCR.
qPCR reagent	SYBR Green Master Mix	Applied Biosystems, Foster City, CA, USA	qPCR on the StepOnePlus Real-Time PCR System.
Transfection reagent	Lipofectamine RNAiMAX	Thermo Fisher Scientific, Waltham, MA, USA	siRNA transfection into LX-2 cells.
Antibody	Anti-C7 antibody	Sigma-Aldrich/Merck KGaA; HPA001465; rabbit polyclonal	Double immunofluorescence on F4 liver tissue.
Antibody	Anti-alpha-SMA antibody	Dako/Agilent Technologies; clone 1A4; M0851; mouse monoclonal	Detection of activated hepatic stellate cell/myofibroblast areas.
ISH probe	RNAscope Target Probe - Hs-C7	Cosmo Bio, Tokyo, Japan; ADC534791	In situ hybridization for C7 transcripts in liver tissue.

3. Oligonucleotides and cell experiments

Category	Resource / item	Sequence / condition	Use / key details
Primer	C7 forward	5'-GGCTGGCTGCGTGTTCTTGA-3'	qPCR target: C7.
Primer	C7 reverse	5'-CAGGAGTTGGAGACGAGCAT-3'	qPCR target: C7.
Primer	PLCXD3 forward	5'-TTCCATGAATAAGATGGAGAAAGCTCATTG-3'	qPCR target: PLCXD3.
Primer	PLCXD3 reverse	5'-GTACCTTACCATTTACTGGGTACTTGCA-3'	qPCR target: PLCXD3.
Primer	NALCN forward	5'-ACTGACTTTGGTCCTGATGA-3'	qPCR target: NALCN.
Primer	NALCN reverse	5'-ACTGAAGTGGAGGATAGTGC-3'	qPCR target: NALCN.
Primer	LAMA2 forward	5'-ACGCTGAGAAACTTGAGGCCA-3'	qPCR target: LAMA2.
Primer	LAMA2 reverse	5'-TCAATATCTTTGAGGCAGCCGGA-3'	qPCR target: LAMA2.
Primer	LAMB1 forward	5'-CGCACCGCACGAGAGTTT-3'	qPCR target: LAMB1.
Primer	LAMB1 reverse	5'-CGCATTTACGTTCGCAAGAA-3'	qPCR target: LAMB1.
Primer	COL6A3 forward	5'-CCTAACCACATATGTTAGTGGAGGT-3'	qPCR target: COL6A3.
Primer	COL6A3 reverse	5'-GAATGTCTCGCTTGCTCTCTG-3'	qPCR target: COL6A3.
Primer	GAPDH forward	5'-GCTTCGCTCTCTGCTCCT-3'	qPCR internal control.
Primer	GAPDH reverse	5'-CGCCCAATACGACCAAAT-3'	qPCR internal control.
Cell line	LX-2 cells	Human hepatic stellate cell line; Sigma-Aldrich	siRNA-mediated C7 knockdown experiment.
Cell culture	LX-2 maintenance	DMEM + 10% FBS + 1% penicillin/streptomycin; 37°C; 5% CO_2	Routine culture before transfection.
Transfection	C7 siRNA and non-targeting control siRNA	Final concentration 30 nM; harvested on day 3	Assessment of knockdown efficiency and ECM-related gene expression.

4. Instruments, platforms, datasets, and software

Category	Resource / item	Source / identifier	Use / key details
Instrument	FibroScan	Echosens, Paris, France	VCTE for LSM and CAP; FAST score calculation.
Instrument	StepOnePlus Real-Time PCR System	Applied Biosystems, USA	qPCR data acquisition.
Sequencing platform	Illumina NovaSeq 6000	Illumina, San Diego, CA, USA	Sequencing of spatial transcriptomic libraries.
Spatial transcriptomics	10x Genomics platform	10x Genomics, Pleasanton, CA, USA	Spatially barcoded arrays and library preparation.
Public dataset	GSE49541	Gene Expression Omnibus	Hepatic microarray transcriptomic dataset for fibrosis-stage DEG analysis.
Public dataset	GSE174748	Gene Expression Omnibus; Seurat object	snRNA-seq dataset including NASH/MASLD-cirrhosis and healthy liver tissue.
Proteomics database	Proteome-Phenome Atlas	proteome-phenome-atlas.com	Plasma proteomic data for proteins associated with liver cirrhosis/fibrosis.
Bioinformatics resource	GEO2R	NCBI GEO web tool	Differential expression analysis of GSE49541.
Bioinformatics resource	Metascape, GO, KEGG, Reactome	Web resources / pathway databases	Functional enrichment and network visualization of C7-associated genes.
Software	StatFlex	Version 7.0	Statistical analyses.
Software	R	Version 4.3.0	Statistical analysis and bioinformatics workflows.
R package	limma	Bioconductor	Differential gene/protein expression; Benjamini-Hochberg FDR correction.
R package	Seurat	Version 5.1.0	snRNA-seq quality control, normalization, scaling, and downstream analysis.
R function	geom_violin()	ggplot2 / R ecosystem	Violin plot visualization of C7 expression.
Software	GraphPad Prism	Version 10	Graphing and statistical analyses.
Software	SpaceRanger and Loupe Browser	10x Genomics	Processing, visualization, and spatial gene expression analyses.

5. Key analytical methods and outcome definitions

Category	Resource / item	Identifier / condition	Use / key details
Histology	NAFLD activity score	Steatosis 0-3; lobular inflammation 0-3; ballooning 0-2; NAS 0-8	Assessment by an independent blinded pathologist.
Histology	Fibrosis staging	F0-F4	Assessment of fibrosis severity.
Clinical outcome	LRE in follow-up cohort	HCC, grade II or higher hepatic encephalopathy, poorly controlled ascites requiring hospitalization, and varices requiring endoscopic treatment including rupture	Incident liver-related event outcome during observation.
Clinical outcome	LRE status in VCTE cohort	Current or prior LREs at MASLD diagnosis	Cross-sectional validation outcome.
External outcome	MALO in UK Biobank	Ascites, cirrhosis, varices, HCC, liver failure, liver transplantation, and other major liver outcomes by ICD-10 codes	External outcome for UK Biobank validation.
VCTE protocol	LSM	At least 10 valid measurements; IQR/median <=30%; fasting state	Median LSM used for analysis.
qPCR method	Relative gene expression	Delta-delta Ct method; GAPDH internal control; 95°C 10 min, 40 cycles of 95°C 15 sec and 60°C 1 min	Analysis of liver biopsy and LX-2 cell gene expression.
Spatial module score	ECM/HSC-related fibrogenic score	ACTA2, COL1A1, COL1A2, COL3A1, COL6A3, LAMA2, LAMB1, TAGLN, PDGFRB, DCN	Average normalized expression after z-score standardization per spatial spot.
Spatial module score	Hypoxia score	CA9, VEGFA, ADM, PDK1, LDHA, ENO1, HK2, PGK1, EGLN3	Average normalized expression after z-score standardization per spatial spot.
Spatial module score	Progenitor/ductular reaction score	SOX9, PROM1, KRT19, EPCAM, TACSTD2, KRT7	Average normalized expression after z-score standardization per spatial spot.
ISH scoring	Five-grade RNAscope scoring system	Score 0-4; score 0 negative; scores 1-4 positive	Semi-quantitative evaluation of C7 mRNA expression.
snRNA-seq QC	Nuclear filtering	nCount_RNA or nFeature_RNA outside 0.5-99.5% quantile range, or percent.mito >10%, excluded	Filtering of GSE174748 nuclei.
snRNA-seq processing	Normalization and scaling	Seurat LogNormalize(); scaled data	Analysis of C7 expression across liver cell types.
snRNA-seq annotation	HSC annotation	Mesenchymal cells reclassified as HSCs	Focused analysis of HSC-associated C7 expression.
UK Biobank MASLD definition	MASLD criteria	FLI >=60 plus at least one cardiometabolic risk factor and alcohol intake within MASLD threshold	Eligibility definition for external validation.
UK Biobank alcohol estimation	Ethanol conversion	Alcohol (g) = volume (mL) x alcohol-by-volume decimal x 0.789	Conversion of self-reported beverage intake to g/day.
Statistical analysis	Group comparisons, correlations, regression, survival, and ROC analyses	Mann-Whitney U test, Kruskal–Wallis test, chi-square test, Fisher's exact test, Spearman correlation, multiple linear regression, Cox regression, Kaplan-Meier/log-rank, ROC/AUROC, Youden index	Primary statistical methods used in the study.
Data availability	Public and controlled-access data	GSE49541; GSE174748; Proteome-Phenome Atlas; UK Biobank application no. 930515	Public datasets and controlled-access external validation resource.
